# Synthesis and antimycotic activity of new derivatives of imidazo[1,2-*a*]pyrimidines

**DOI:** 10.3762/bjoc.20.236

**Published:** 2024-11-05

**Authors:** Dmitriy Yu Vandyshev, Daria A Mangusheva, Khidmet S Shikhaliev, Kirill A Scherbakov, Oleg N Burov, Alexander D Zagrebaev, Tatiana N Khmelevskaya, Alexey S Trenin, Fedor I Zubkov

**Affiliations:** 1 Organic Chemistry Department, Voronezh State University, 1 Universitetskaya pl., 394018 Voronezh, Russian Federation; 2 Laboratory of Bio- and Cheminformatics, HSE University, 194100 St. Petersburg, Russian Federation; 3 Department of Chemistry, Southern Federal University, 7 R. Zorge St., 344090 Rostov-on-Don, Russian Federation; 4 The Smart Material Southern Federal University, Southern Federal University, 178/24 Andrei Sladkova St., 344090 Rostov-on-Don, Russian Federation; 5 Clinical Laboratory Diagnostics Department, N. N. Burdenko Voronezh State Medical University, 10 Studencheskaya St., 394036 Voronezh, Russian Federation; 6 Gause Institute of New Antibiotics, 11 B. Pirogovskaya St., 119021 Moscow, Russian Federation; 7 Organic Chemistry Department, RUDN University, 6 Miklukho-Maklaya St., 117198 Moscow, Russian Federation

**Keywords:** 2-aminoimidazole, antimycotic activity, imidazo[1,2-*a*]pyrimidine, molecular docking, *N*-arylitaconimides, *N-*substituted maleimides, recyclization

## Abstract

The heterocyclic core of imidazo[1,2-*a*]pyrimidine was formed in satisfactory yields as a result of the interaction of the readily available 2-aminoimidazole with *N*-substituted maleimides or *N*-arylitaconimides. The mechanism of the studied processes was postulated basing on experimental data, HPLC–MS analysis of reaction mixtures, and quantum chemical calculations. Molecular docking results of the obtained imidazo[1,2-*a*]pyrimidines, when compared with voriconazole, a drug already in clinical use, suggest that they may possess antifungal activity against *Candida albicans*.

## Introduction

**Figure 1:**
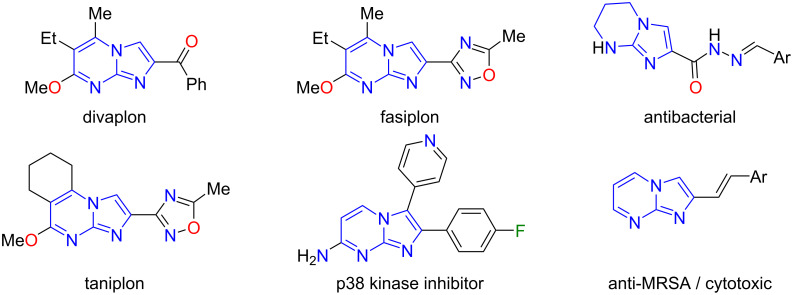
Some biologically active compounds and organic fluorophores containing the imidazo[1,2-*a*]pyrimidine nucleus.

Nitrogen-containing heterocyclic compounds occupy a pivotal position in the arsenal of modern organic and medicinal chemistry due to their extensive spectrum of physiological activity [1]. Imidazo[1,2-*a*]pyrimidines represent one of the most promising classes of compounds within this group [2–3]. These small azoheterocyclic frameworks (small-molecule concept) offer a distinctive advantage in the synthesis of new biologically active molecules, as they are synthetic bioisosters of purine bases. Imidazo[1,2-*a*]pyrimidine derivatives exhibit a wide range of pharmacological properties [4–6]. For example, this scaffold is a key structural element of divaplon [7], fasiplon and taniplon [8], which are anxiolytics and anticonvulsant drugs. However, their application in clinical practice was recently discontinued [9–10]. The use of imidazo[1,2-*a*]pyrimidine derivatives as effective antifungal agents is worthy of particular attention, as evidenced by the following references [11–15] (Figure 1).

**Figure 2:**
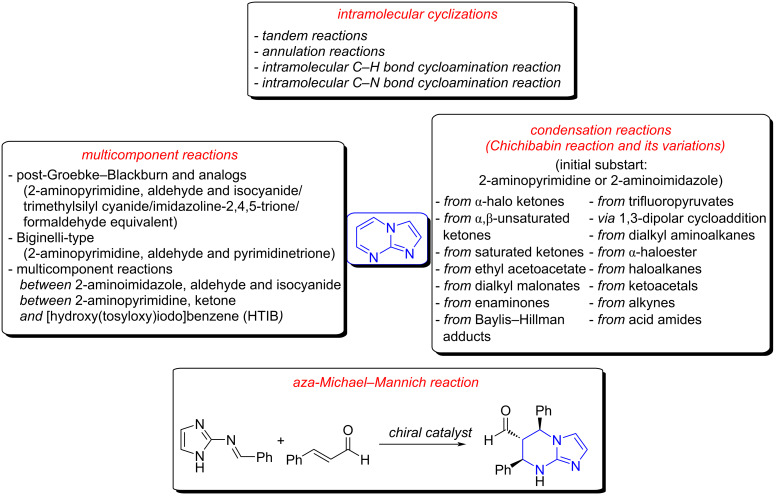
Existing approaches to imidazo[1,2-*a*]pyrimidines.

The best known method for the synthesis of imidazo[1,2-*a*]pyrimidines is the one developed by Chichibabin [16–19], based on the reaction between 2-aminopyrimidine and α-haloketones. Despite the widespread use of imidazo[1,2-*a*]pyrimidines obtained by various modifications of this method and a number of others described in detail in the review by Goel et al. [4], literature data on the synthesis of tetrahydroimidazo[1,2-*a*]pyrimidines without substituents in the second and third positions remain limited. In this context, it is worth mentioning the work of Li and co-workers (2011) [20], who described a single example of the formation of such structures by carrying out an organocatalytic domino aza-Michael–Mannich reaction between benzylidene-1*H*-imidazol-2-amine and cinnamaldehyde. Although the imidazo[1,2-*a*]pyrimidines thus formed did not show significant bioactivity, they found applications as additives in electrochemical copper plating processes [21–22] (Figure 2).

Based on the above, the main goal of this work was to develop a convenient method for the construction of potentially pharmacophoric imidazo[1,2-*a*]pyrimidines using readily available derivatives of azaheterocycles as starting materials. As the last ones, we chose the reaction between 2-aminoimidazole and *N*-arylitaconimides or *N-*substituted maleimides, which can be analyzed by both classical synthetic and quantum DFT methods. The second problem solved in this work was a preliminary evaluation of the potential bioactivity of the obtained compounds. In particular, a molecular docking experiment to investigate the binding mechanisms to the CYP51 enzyme and an evaluation of the antifungal activity of imidazo[1,2-*a*]pyrimidines against *Candida albicans* were performed.

## Results and Discussion

*N*-Arylitaconimides [23] and *N-*substituted maleimides [24] were used as initial reagents in the synthesis of the target tetrahydroimidazo[1,2-*a*]pyrimidine derivatives. These compounds are promising sources of C3-synthons and offer the possibility of constructing polysubstituted hydrogenated heterocyclic structures on their basis [25–28], including an acetanilide fragment. The introduction of this fragment into a molecule, often drugs, enhances the cytotoxic, antibacterial, and antiviral activity of the compounds, thus widening the range of their potential applications [28–29] including as antifungal agents [30–33]. These findings are supported by a number of literature sources that highlight the importance of incorporating the acetanilide moiety into potential drugs, including for enhancing the pharmacological activity of imidazo[1,2-*a*]pyrimidines [1].

It has already been noted that the preferred medium for recyclizations involving imides of itaconic and malic acids by various *N,N*- and *C,N*-dinucleophiles, in particular by 1,2-diaminoimidazole derivatives, 5-aminopyrazoles, and 3-aminocyclohexen-2-ones, are mixtures of polar solvents with acetic acid or pure acetic acid [25–28,34–36].

**Scheme 1:**
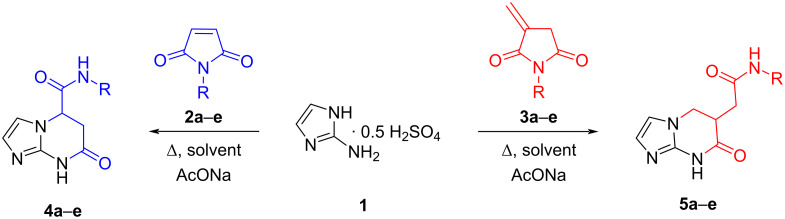
Reaction of 2-aminoimidazole (**1**) with *N-*substituted maleimides (**2**) and *N*-arylitaconimides (**3**).

Within the framework of this study, the primary task was to establish the optimal reaction conditions for the interaction of 2-aminoimidazole (**1**) with *N-*phenylmaleimide (**2a**) (Scheme 1). The choice of the investigated reaction conditions was based on the analysis of existing data [28], taking into account experimentally obtained data on the solubility of the starting reagents. The tested solvents were dioxane, PhMe, MeOH, EtOH, iPrOH, MeCN, and DMF (dimethylformamide). Sodium acetate as an additive was necessary for converting 2-aminoimidazole hemisulfate to its basic form in situ. Thin-layer chromatography (TLC) and high-performance liquid chromatography with mass-spectrometric detection (HPLC–MS) were used to monitor the reaction and to identify the products obtained. It should be noted that in the case of HPLC–MS analysis, the signals registered in the mass spectra were interpreted on the basis of pre-calculated weights (as molecular ions with [M + H]^+^) for all possible initial, intermediate, and expected interaction products (Table 1).

**Table 1:** Screening of reaction conditions for the preparation of 7-oxo-*N*-phenyl-5,6,7,8-tetrahydroimidazo[1,2-*a*]pyrimidine-5-carboxamide (**4a**).

Entry	Solvent and additives	Time of the reaction, h				
		0.5	1	2	3	12
		Amount (%) of **4a** in the reaction mixture				

1	PhMe, AcONa (1 equiv)	2	12	18	22	25
2	dioxane, AcONa (1 equiv)	5	15	24	30	35
3	DMF, AcONa (1 equiv)	10	10	10	10	10
4	MeCN, AcONa (1 equiv)	12	15	19	19	19
5	iPrOH, AcONa (1 equiv)	20	45	58	65	65
6	MeOH, AcONa (1 equiv)	8	27	42	48	48
7	EtOH, AcONa (1 equiv)	12	31	46	58	58
8	iPrOH, AcONa (1.5 equiv)	39	57	72	72	72
**9**	**iPrOH, AcONa (2 equiv)**	60	**89**	89	89	89
10	iPrOH, AcONa (2.5 equiv)	57	85	85	85	85

The tentative experiments showed that when toluene or dioxane were used as solvents, the maximum conversion of the reagents was not achieved until after 12 h of boiling, and the yield of the product varied in the range of 25–35% (entries 1 and 2, Table 1). In the cases of DMF and MeCN, the formation of complex, inseparable mixtures of numerous intermediates and products of their subsequent intramolecular cyclization were observed (Table 1, entries 3 and 4).

The best results were obtained when isopropyl alcohol was used, which furnished yields of the final product **4а** up to 89% within 1 h (entry 9 in Table 1). In the case of methanol and ethanol, the maximum conversion was also observed during the three-hour boiling, but the yields of the product **4a** were significantly lower. It is important to note that the amount of sodium acetate introduced had a significant effect on the rate and completeness of the conversion of the starting materials to the final product (see as an example, entries 8 and 9 in Table 1).

The optimal conditions found for product **4a** also proved to be efficient for the preparation of its analogs **5** from *N*-arylitaconimides **3** (Scheme 1). Therefore, a broad range of variously substituted imidazo[1,2-*a*]pyrimidines was synthesized (Table 2).

**Table 2:** Yields of the products **4** and **5**.

Entry	Initial imide	R	Product	Yield, %

1	**2a**	Ph	**4a**	89
2	**2b**	4-iPrC_6_H_4_	**4b**	84
3	**2c**	2-Me,3-ClC_6_H_3_	**4c**	86
4	**2d**	2,5-Cl_2_C_6_H_3_	**4d**	83
5	**2e**	2-Me,5-NO_2_C_6_H_3_	**4e**	82
6	**2f**	CH_2_C_6_H_5_	**4f**	84
7	**2g**	CH_2_CH_2_C_6_H_5_	**4g**	75
8	**2h**	CH_2_CH_2_(4-MeОC_6_H_4_)	**4h**	89
9	**2i**	Me	**4i**	70
10	**3a**	Ph	**5a**	92
11	**3b**	4-BrC_6_H_4_	**5b**	86
12	**3c**	4-FC_6_H_4_	**5c**	83
13	**3d**	4-ClC_6_H_4_	**5d**	87
14	**3e**	4-EtC_6_H_4_	**5e**	88

**Scheme 2:**
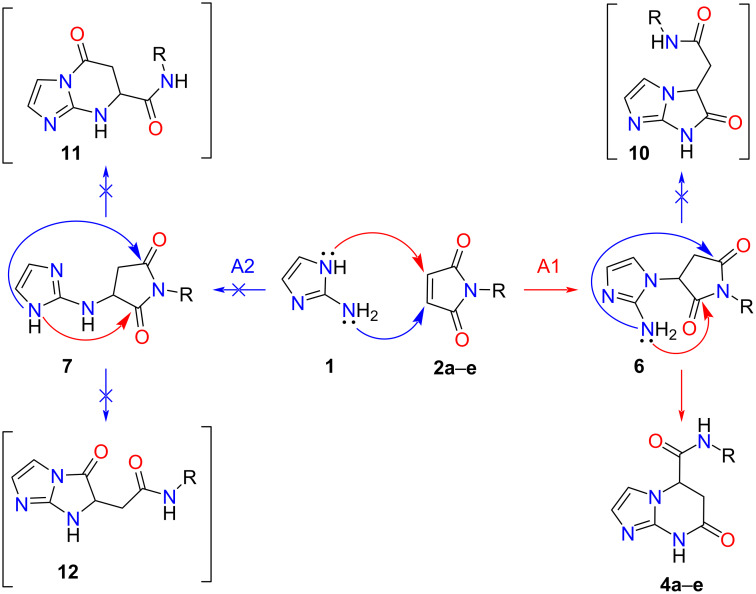
Plausible synthetic routes for the interaction of *N*-substituted maleimides **2** with 2-aminoimidazole (**1**).

**Scheme 3:**
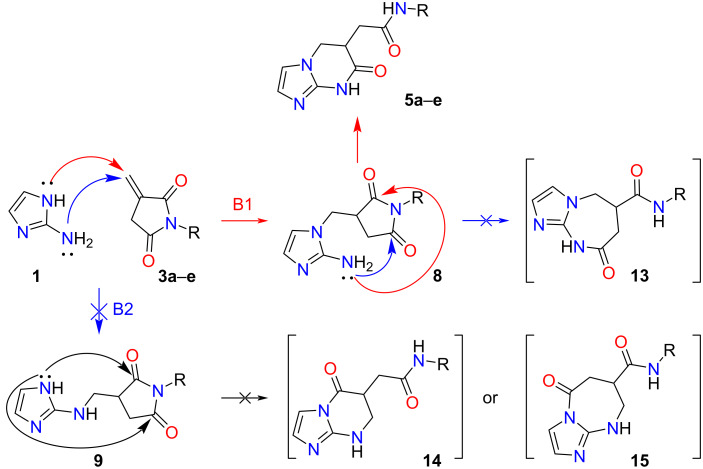
Plausible synthetic routes for the interaction of or *N*-arylitaconimides **3** with 2-aminoimidazole (**1**).

For the interaction of the polyfunctional precursors **1**, **2**, and **3**, two pathways are theoretically possible (Scheme 2 and Scheme 3). The first one involves the *N*-nucleophilic Michael addition to the activated multiple bond of the imide, leading to the formation of linear intermediates **6** or **8** (pathways A1 and B1) at the expense of the *endo*-nitrogen atom. The second route involves the participation of an amino group which allows the formation of adducts **7** and **9** (routes A2 and B2).

The intermediates formed then undergo subsequent tandem recyclization of succinimide/citraconimide fragments at the expense of one of the carbonyl groups and the imidazole nucleophilic center not involved in the first step. This process leads to the formation of alternative final products: imidazo[1,2-*a*]imidazoles **10** and **12**, imidazo[1,5-*a*]pyrimidines **4**, **5**, **11** and **14**, and imidazo[1,2-*a*]diazines **13** and **15**.

The analysis of the spectral data (^1^H and ^13^C NMR, 2D NMR spectroscopy, HPLC–HRESIMS) of the products of the investigated reactions unambiguously confirms the processes proceeding along routes **A1** and **B1** with the formation of *N*-aryl(alkyl)-7-oxo-5,6,7,8-tetrahydroimidazo[1,2-*a*]pyrimidin-5-carboxamides **4a**–**i** and *N*-aryl-2-(7-oxo-5,6,7,8-tetrahydroimidazo[1,2-*a*]pyrimidin-6-yl)acetamides **5a**–**e**.

In ^1^H NMR spectra of the products, the characteristic signals for the protons of the methylene and methine fragments of the pyrimidine cycle are important in establishing the regiochemistry of the process. Thus, in the ^1^Н NMR spectra of compounds **4**, the following reference signals of protons are present: H_А_-6 (d at δ ≈ 2.70–2.80 ppm, ^2^*J*_6А,6В_ = 16.6 Hz and ^3^*J*_5,6А_ = 2.8 Hz), H_В_-6 (dd at δ ≈ 3.20–3.30 ppm, ^2^*J*_6А,6В_ = 16.6 and ^3^*J*_5,6В_ = 7.5 Hz), and H-5 (δ = 5.05–5.25 ppm, ^3^*J*_5,6В_ = 7.5 Hz ^3^*J*_5,6А_ = 2.8 Hz) [37]. It is worth noting that in the spectra of the supposed compounds **11**, the HB-5 proton would undergo additional cleavage at the proton of the adjacent NH group of the pyrimidine ring. Additionally, in the probable structures **10** and **12** the protons HA-5 and HA-6 should have been magnetically equivalent. The same reasoning can be applied to the spectra of **5a**−**e**.

**Figure 3:**
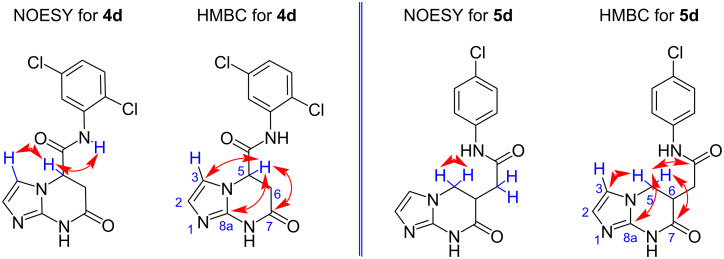
Key correlations observed in the NOESY and HMBC spectra of the products **4d** and **5d**.

The unambiguous assignment of the signals for the methine and methylene groups of compounds **4** and **5** was carried out based on the correlations found in the NOESY ^1^H,^1^H and HMBC ^1^H,^13^C spectra. As an example, the key correlation interactions for compounds **4d** and **5d** are depicted in Figure 3.

Thus, in the NOESY spectra of the imidazopyrimidine **4d** there are cross peaks of the methine proton at C-5 with the amide proton of the acetamide fragment (Figure 3), which are not possible in the case of the hypothetical imidazoimidazoles **10** and **12**. Also the observed cross peak of the same methine proton with the proton at C-3 of the imidazole core, would not be possible for the alternative imidazopyrimidine system. These results allowed us to reject the structure **11**. The lack of correlation in the HMBC spectra between the protons of the amide fragment and the C-5 carbon atom, as well as the presence of cross-peaks of the H-5 proton with the C-8a nodal atom and the C-3 imidazole carbon atom, further supports the formation of product **4d**.

Strong interactions between protons of two methylene groups are the most noticeable features in the NOESY spectrum of the imidazo[1,2-*a*]pyrimidine **5d**. Due to the conformational rigidity of its heterocyclic core, only geminal interactions are observed. Hence, the cross peaks can be observed only between two protons of the *exo*-5-methylene group and between two protons of the *endo*-methylene group (see Figure 3). Vicinal proton interactions are weakly expressed in the NOESY spectra and the absence of cross peaks between the methine proton and the endocyclic methylene group is apparently due to a conformational effect. Significantly, there are no cross peaks for the protons of the *endo*-methylene group with the NH proton of the pyrimidine cycle, which would be observed in the alternative structures.

In the HMBC spectrum of the compound **5d**, the cross peaks of the *endo*-methylene protons (CH_2_-5) with the carbons C-3 and C-8a are the most informative, indicating a close proximity of the interacting nucleus that cannot be realized in the alternative structures (for example, **14** and **15** in Scheme 3). In addition, clearly distinguishable cross-peaks between the protons of the CH_2_-5 group and both *endo* and *exo*-carbonyl groups are observed. This allowed us to explicitly exclude from the analysis structures **13** and **15**, which contain both *endo*-methylene groups, as part of a conformationally rigid seven-membered cycle. In the latter case, the picture would be different: the interaction of both methylene groups with the carbonyl carbon atom would be observed to an equal extent.

**Scheme 4:**
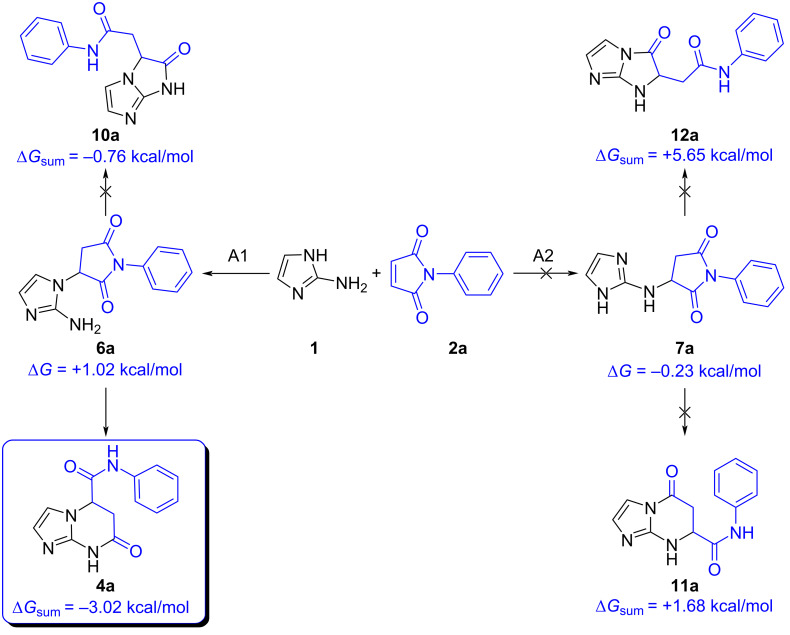
Results of MEP calculations for the reaction of *N*-phenylmaleimide (**2a**) with 2-aminoimidazole (**1**).

**Scheme 5:**
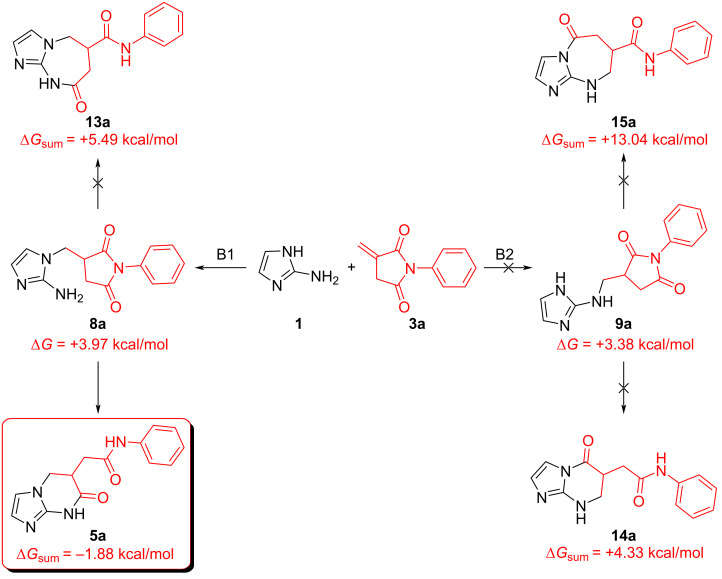
Results of MEP calculations for the reaction of *N*-phenylithaconimide (**3a**) with 2-aminoimidazole (**1**).

In order to obtain additional information on the course of the reaction between 2-aminoimidazole **1** and imides **2** and **3**, the minimum energy paths (MEPs) of these processes were calculated. Quantum chemical DFT calculations were performed using the B3LYP/6-311++G(d,p) basis set and taking into account solvation effects using the polarizable continuum model (PCM). Interactions of **1** with *N*-phenylmaleimide (**2a**) and *N*-phenylithaconimide (**3a**) were considered as model systems (Scheme 4 and Scheme 5).

Based on the calculations performed, the existence of thermodynamically favourable interaction pathways between aminoimidazole **1** and *N*-phenylmaleimide **2a** (pathway A1 and A2) was confirmed, in which intermediates **6a** and **7a** are formed (Scheme 4). Although intermediate **7a** has a lower activation energy (∆*G* = −0.23 kcal/mol), further recyclization processes are not possible due to the positive free energy change (∆*G* > 0). In this context, the formation of the final product is only possible to proceed via intermediate **6a**, which undergoes subsequent cyclization steps more favorably, leading to the formation of the target product **4a** (∆*G* = −3.02 kcal/mol). This suggests that the first step of intermediate formation is the critical one.

It is also noteworthy that the Michael addition via intermediate **6a** is an irreversible process, given that it is thermodynamically challenging to revert the formed target product **4a** to the original starting substances or the corresponding intermediate **6a** (∆*G*(**4a**→**6a**) = + 4.04 kcal/mol). Meanwhile, a minimal difference in the free energy of intermediates **6a** and **7a** may facilitate their interconversion into each other.

A peculiarity of the reaction with *N*-phenylithaconimide (**3a**) is that the formation of intermediates **8a** and **9a** is an endothermic process (paths B1 and B2, correspondingly, Scheme 5). However, the formation of the final product from the starting reagents (∆*G* = −1.88 kcal/mol) as well as from intermediate **8a** (∆*G* = +3.97 kcal/mol) is thermodynamically more favorable than for all other routes (path B).

Fungal infections are a growing threat to public health, resulting in significant economic costs to healthcare systems [38–39]. One of the most common fungal diseases is invasive candidiasis caused by species of the genus *Candida* [40–41], including *C. albicans*, *C. glabrata*, *C. tropicalis,* and *C. parapsilosis*. Bloodstream infections caused by these pathogens are associated with high morbidity and mortality, particularly in intensive care patients. Risk factors for invasive candidiasis include the use of immunosuppressive and cytotoxic drugs, broad-spectrum antibiotic therapy, pre-existing illnesses such as AIDS or diabetes, and the use of central venous catheters, urethral catheters, and implantable medical devices [42].

The mainstay of therapy for these infections is the use of broad-spectrum antifungal agents. Among the many drugs and hits, the azole-based systems (including triazoles and imidazoles) are of particular importance in this context. Their mechanism of action is the inhibition of the activity of the enzyme lanosterol 14α-demethylase (CYP51), which is encoded by the CYP51/ERG11 gene. This enzyme contains a haem-like prosthetic group in its active center and is a member of the cytochrome P450 family, which plays a key role in the biosynthesis of sterols. Sterols in turn are integral components of the fungal cell membrane, making inhibition of CYP51 [43] an effective method of controlling fungal infections. The mechanism of inhibition of azoles and their derivatives is based on the formation of a coordination bond between their heterocyclic nitrogen atom, which carries an unshared electron pair, and the haem iron atom. The formation of this bond leads to inhibition of the catalytic activity of CYP51.

A number of previous studies confirmed the efficacy of azole compounds and their derivatives against a variety of strains and clinical isolates of the genus *Candida*. Nevertheless, the increased use of azole antifungals for the therapy of both mucosal and systemic *Candida* infections has favored the selection and/or emergence of *Candida* strains with advanced resistance in patients suffering from life-threatening disseminated forms of the disease, especially in the presence of serious comorbidities or immunodeficiency. The emergence of resistance to azoles [44–46] may prove to be a significant clinical problem, highlighting the importance of finding alternative therapies for fungal infections. Although there are publications on the use of imidazo[1,2-*a*]pyrimidine derivatives as antifungal agents [47–49], these compounds have not yet demonstrated the desired pharmacological properties. Therefore, the design and synthesis of new compounds of this class with improved ADMET (absorption, distribution, metabolism, excretion, toxicity) pharmacological properties is an urgent task.

**Figure 4:**
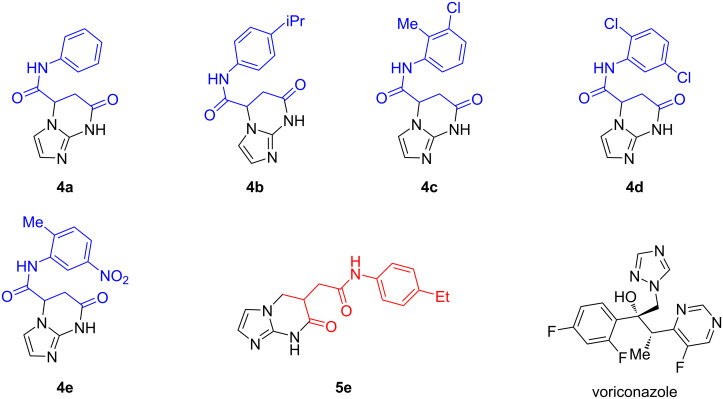
Structures of imidazo[1,2-*a*]pyrimidines selected for docking and voriconazole selected for comparison.

Molecular docking was carried out to identify potential binding positions of compounds **4a**–**e** and **5a**–**e** to CYP51 and to evaluate which of these molecules could act as inhibitors of the enzyme. As mentioned above, CYP51 inhibitors contain a heterocyclic nitrogen atom that forms a coordination bond with haem iron. Therefore, only those compounds that could form such a bond according to the molecular docking results were selected as potentially active. Such compounds were the (*S*)-isomers of compounds **4a**–**e** and the (*R*)-isomer of compound **5e** (Figure 4). No suitable docking solutions were found for the remaining compounds **5a**–**d** in both isomers, the (*R*)-isomers of compounds **4a**–**e** and the (*S*)-enantiomer of **5e** and therefore, these compounds were classified as inactive.

The docking results are shown in Table 3. Compounds **4a**–**e** bind to the active site of CYP51 with affinities ranging from −7.7 to −8.8 kcal/mol and compound **5e** has a much higher affinity of −5.4 kcal/mol. Therefore, we assume that the inhibitor conformation found by docking is energetically unfavorable and therefore the expected activity of compound **5e** will be much lower than that of compounds **4a**–**e**.

**Table 3:** Characterization of ligand–protein interactions for voriconazole and compounds selected during docking.^a^

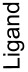	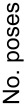	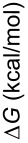	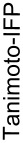	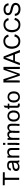	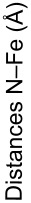	Ligand–protein interactions											

						Y122	F130	V135	Y136	A307	I373	L508	HEM	H374	I377	F504	F234
						H^b^	H	H	H	H	H	H	H/MA^b^	H	H	H	H

**V**	−	−	1	1	2.1	+	+	+	+	+	+	+	+/+	−	−	−	−
**4b-** ***S***	2	−8.3	0.636	0.426	2.9	+	+	−	+	+	+	+	+/+	+	+	+	−
**4c-** ***S***	2	−7.9	0.6	0.493	3.4	+	+	−	−	+	+	+	+/+	+	−	+	−
**4a-** ***S***	3	−8	0.667	0.424	3.5	+	+	−	−	+	+	+	+/+	−	−	+	−
**4d-** ***S***	2	−7.7	0.667	0.478	3.5	+	+	−	−	+	+	+	+/+	−	−	+	−
**4e-** ***S***	1	−8.8	0.667	0.416	2.8	+	+	−	−	+	+	+	+/+	−	−	+	−
**5e-** ***R***	18	−5.4	0.417	0.478	2.5	+	−	−	−	+	+	+	+/+	+	+	+	+

^a^Tanimoto IFP: Tanimoto coefficient for ligand–protein interaction fingerprints. Tanimoto MACCS: Tanimoto coefficient for chemical structure similarity calculated from MACCS descriptors. Hydrophobic – hydrophobic interaction. Metal acceptor – in this case the coordination bond between haem iron and heterocyclic nitrogen. "+": interaction present, "−": absent; ^b^H: hydrophobic, MA: metal acceptor.

Molecular docking data can therefore be used to infer the structure–activity relationship. The substituent on the C-5 atom of tetrahydroimidazo[1,2-*a*]pyrimidine favors the inhibitory activity of this class of compounds, whereas the position of the substituent on the C-6 atom disrupts it. Furthermore, only the (*S*)-isomers of the compounds under consideration are expected to be potent inhibitors of CYP51.

The analysis of the interaction fingerprints (IFP) between the docking ligands and the protein shows that, similar to the reference ligand voriconazole, the compounds interact with the protein through hydrophobic interactions with hydrophobic residues of the protein and the formation of coordination bonds with the haem iron (Table 3). At the same time, hydrogen and ionic bonds are not observed for either the tested compounds or voriconazole. The Tanimoto coefficient, calculated for the interaction footprints of compounds **4a**–**e** relative to voriconazole, indicates a range of values between 0.60 and 0.67. This suggests that the interaction patterns of these compounds differ by approximately four amino acids. The Tanimoto coefficient for compound **5e** is significantly lower (≈0.42), indicating that the interaction pattern of compound **5e** is significantly different from that of voriconazole, which may explain its low predicted affinity. The Tanimoto coefficient calculated from the MACCS descriptors for the selected compounds is <0.5, indicating that the chemical structure of compounds **4a**–**e** and **5e** is significantly different from voriconazole.

**Figure 5:**
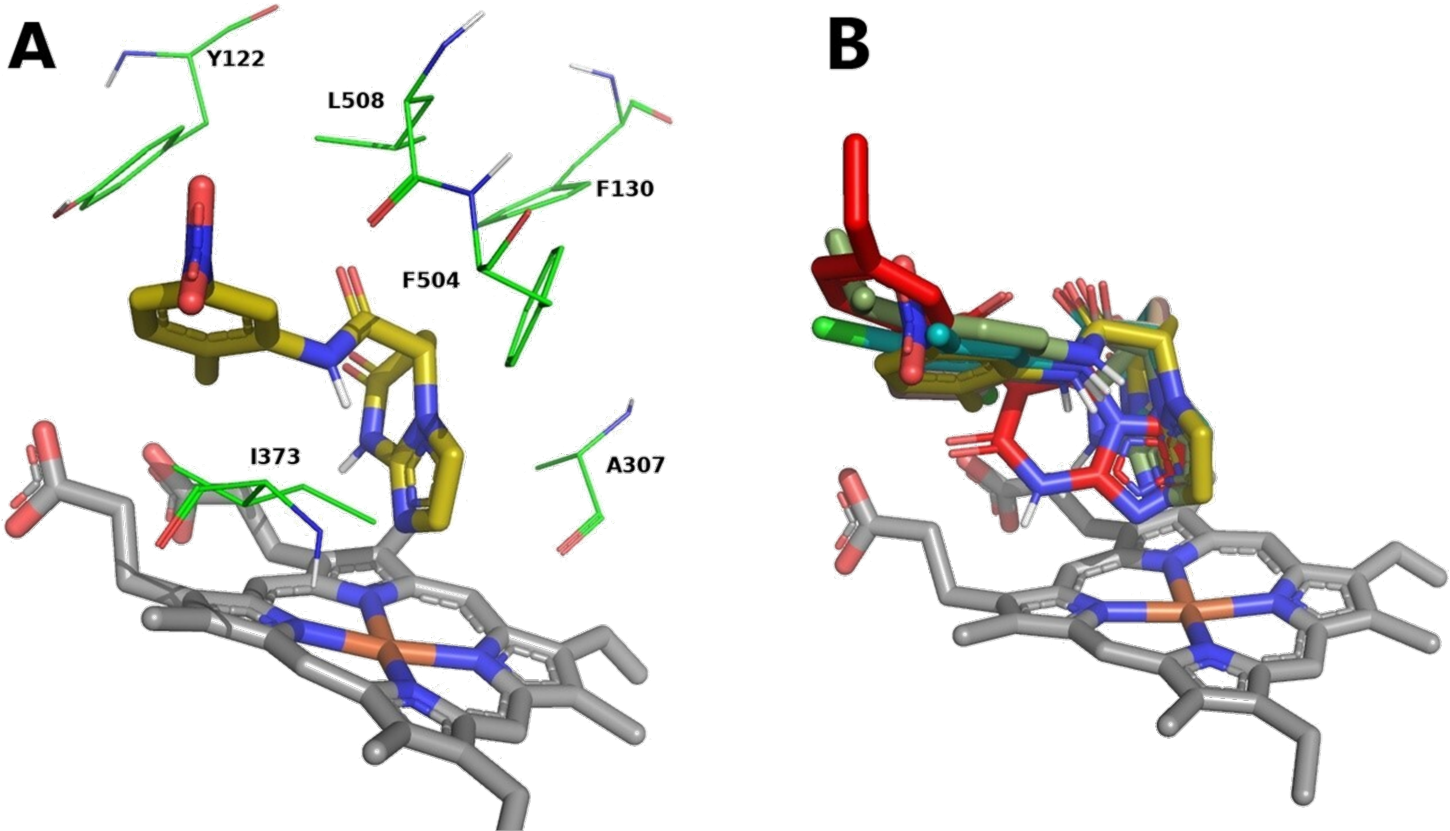
(A) Position of the (*S*)-isomer of compound **4e** in the active site of CYP51 after molecular docking^a^. (B) Superposition of docked compounds **4a**–**e** and **5e** in a CYP51 AC^b^. ^a^The amino acid residues of the protein are colored green, compound **4e** is colored gold and the haem is colored grey. The heterocyclic nitrogen of compound **5e** appears to be orientated towards iron and is located 2.8 Å away from it, which may indicate the formation of a coordination bond; the benzene moiety is located in a hydrophobic pocket. ^b^The conformations of compounds **4a**–**e** are identical, whereas the position of compound **5e** (red) is significantly different.

The three-dimensional position of the selected compounds in the active site of the enzyme is shown in Figure 5A for compound **4e**. The heterocyclic nitrogen is indeed orientated towards the haem iron, with an N–Fe distance of 2.8 Å, which is slightly higher than the distance of 2.1 Å in the crystal complex with the inhibitor voriconazole. The benzene substituent on the C-6 atom is located in a hydrophobic pocket formed by residues Y122, L508, F504 and I373. The superposition of the selected and docked ligands reveals that compounds **4a**–**e** are arranged in an identical manner, whereas the position of compound **5e**, particularly tetrahydroimidazo[1,2-*a*]pyrimidine, is significantly disparate (Figure 5B).

## Conclusion

Thus, we have proposed an efficient way to construct tetrahydroimidazo[1,2-*a*]pyrimidines without substituents at the 4 and 5-positions of the imidazole fragment by the reaction of 2-aminoimidazole with *N*-arylitaconimides and *N-*substituted maleimides. With the aid of DFT calculations, the most probable reaction path of the interaction was suggested. Based on the docking data, *N*-aryl(alkyl)-7-oxo-5,6,7,8-tetrahydroimidazo[1,2-*a*]pyrimidin-5-carboxamides as (*S*)-isomers were shown to be potent inhibitors of CYP51. These results allow us to consider these compounds as potential CYP51 inhibitor candidates for further in vitro and in vivo testing as antifungal prodrugs.

## Experimental

**General procedure for preparation of *N*-R-7-oxo-5,6,7,8-tetrahydroimidazo[1,2-*a*]pyrimidine-5-carboxamides 4a–i:** A mixture of 2-aminoimidazole hemisulfate (**1**, 0.66 g, 5 mmol), *N-*substituted maleimides **2a**–**i** (5 mmol), sodium acetate (0.82 g, 10 mmol), and iPrOH (10 mL) was boiled for 1 h. The resulting precipitate was filtered off, washed with water (2 × 5 mL) and recrystallized from a mixture of DMF/iPrOH 1:2. The title compounds were obtained as white (**4a**–**e**) or pink (**4f**–**i**) solids.

**General procedure for the synthesis of *N*-aryl-2-(7-oxo-5,6,7,8-tetrahydroimidazo[1,2-*a*]pyrimidin-6-yl)acetamides 5a–e:** A mixture of 2-aminoimidazole hemisulfate (**1**, 0.66 g, 5 mmol), *N*-arylitaconimide **3a**–**e** (5 mmol), sodium acetate (0.82 g, 10 mmol), and iPrOH (10 mL) was boiled for 1 h. After cooling to rt, the resulting precipitate was filtered off, washed with water (2 × 5 mL) and recrystallized from a mixture of DMF/iPrOH 1:2. The title compounds **5a**–**e** were obtained as white solids.

## Supporting Information

File 1 General reaction procedures, compound characterization data, copies of NMR and mass spectra for all new products.

## Data Availability

All data that supports the findings of this study is available in the published article and/or the supporting information of this article.
