# Supplementary material for: Synthesis and antimycotic activity of new derivatives of imidazo[1,2-a]pyrimidines
Source: Beilstein J Org Chem. 2024 Nov 5;20:2806–17. doi: 10.3762/bjoc.20.236 (PMC11552434; doi:10.3762/bjoc.20.236)
Supplement: File 1 — General reaction procedures, compound characterization data, copies of NMR and mass spectra for all new products. [file Beilstein_J_Org_Chem-20-2806-s001.pdf]

BEILSTEIN JOURNAL OF ORGANIC CHEMISTRY

Supporting Information

for

Synthesis and antimycotic activity of new derivatives of imidazo[1,2-*a*]pyrimidines

Dmitriy Yu. Vandyshev, Daria A. Mangusheva, Khidmet S. Shikhaliev, Kirill A. Scherbakov, Oleg N. Burov, Alexander D. Zagrebaev, Tatiana N. Khmelevskaya, Alexey S. Trenin and Fedor I. Zubkov

Beilstein J. Org. Chem. **2024,** *20,* 2806–2817. doi:10.3762/bjoc.20.236

General reaction procedures, compound characterization data, copies of NMR and mass spectra for all new products

License and Terms: This is a supporting information file under the terms of the Creative Commons Attribution License (https://creativecommons.org/licenses/by/4.0). Please note that the reuse, redistribution and reproduction in particular requires that the author(s) and source are credited and that individual graphics may be subject to special legal provisions.
The license is subject to the *Beilstein Journal of Organic Chemistry* terms and conditions: (https://www.beilstein-journals.org/bjoc/terms)

Table of contents

1. General information ……………………………………………………..………….…S2

2. Analytical data of products ………………………………………………………...S4

3. NMR and mass spectra of products ……………………………………………..S13

1. General information

The purity of the synthesized compounds was monitored by TLC on silica gel 60 F_{254} plates (Merck) using chloroform, ethyl acetate, methanol or mixtures thereof as eluents. Chromatograms were developed by UV irradiation (254 and 365 nm), by treatment with iodine vapor or by heating the plate. Melting temperatures were determined using a Stuart SMP 30.

^{1}H, ^{13}C and ^{19}F NMR spectra were recorded on JNM-ECA 600 or Bruker Avance Neo 700 spectrometers (on 600 and 700 MHz for ^{1}H; 151 and 176 MHz for ^{13}C; 564 and 659 MHz for ^{19}F) using residual solvent signals (7.26/77.0 ppm for ^{1}H/^{13}C $CDCl_3$ and 2.50/39.5 ppm for DMSO-d_6). $CFCl_3$ was used as an internal standard for ^{19}F NMR [1]. Where necessary, assignments of ^{1}H and ^{13}C signals were made with the aid of COSY, NOESY, HSQC and HMBC 2D NMR spectra.

HPLC analysis was performed on an Agilent 1260 Infinity liquid chromatograph equipped with a UV detector coupled to an Agilent 6230 TOF LC/MS detector (electrospray ionization, capillary voltage 4 kV, fragment voltage 191 V, skimmer voltage 65 V, positive ion detection, Poroshell 120 EC-C18 column, 4.6 m × 150 mm, particle size 2.7 µm, mobile phase - H_2O (40−0%), MeCN (60−100%), linear gradient elution 5 min, flow rate 0.4 mL/min, column temperature 40 °C, sample volume 1.5 µL.

Quantum chemical calculations were performed on molecules in the gas phase using density functional theory with the 6-31G** basis set and the B3LYP functional including the three-parameter Beck exchange functional and the Lee-Yan-Parr correlation functional [2].

The structure of A. fumigatus CYP51 (PDB ID: 4UYM [3]) in complex with the antifungal drug voriconazole was used as a target for molecular docking. Compounds **4a−e** and **5a−e** were generated as *R*- and *S*-isomers in the molecular editor package SYBYL and

minimized using Gasteiger-Hückel charges. Docking was performed into the pre-minimized structure of CYP51 with non-protein atoms removed except for haem. Vina Autodock software was used for docking.

The docking poses obtained were selected based on the position of the heterocyclic nitrogen atom relative to the haem iron atom. Those compounds that showed a hypothetical possibility of forming a coordination bond with the haem iron were marked as potentially active.

The interaction fingerprints (IFP) between voriconazole and selected compounds with protein were calculated using the Python library ProLif [4]. In view of the inability of docking to account for coordination bonds due to the required significant overlap of van der Waals radii of atoms, the distance cut-off for the formation of the 'metal-acceptor' interaction type was increased from the ProLif default of 2.8 Å to 3.5 Å. To assess the similarity of chemical structures between voriconazole and selected compounds, Tanimoto coefficients were calculated based on MACCS descriptors (Tanimoto MACCS). To assess the similarity of ligand-protein interactions, Tanimoto coefficients were also calculated for interaction fingerprints (Tanimoto IFP). Tanimoto coefficients were calculated using the RDKit library.

References

1. Rosenau, C.P.; Jelier, B.J.; Gossert, A.D.; Togni, A. *Angew. Chem. Int. Ed.* **2018**, *57*, 9528–9533. https://doi.org/10.1002/anie.201802620

2. Becke, A.D. *Phys. Rev. A. Gen. Phys.* **1988**, *38*, 3098−3100. https://doi.org/10.1103/physreva.38.3098.

3. Hargrove, T.Y.; Wawrzak, Z.; Lamb, D.C.; Guengerich, F.P.; Lepesheva, G.I. *J. Biol. Chem.* **2015**, *290*, 23916−23934. https://doi.org/10.1074/jbc.M115.677310.

4. Bouysset, C.; Fiorucci, S. *J. Cheminform.* **2021**, *13*, 72. https://doi.org/10.1186/s13321-021-00548-6

2. Analytical data of products

7-Oxo-N-phenyl-5,6,7,8-tetrahydroimidazo[1,2-a]pyrimidine-5-carboxamide ***(4a).***

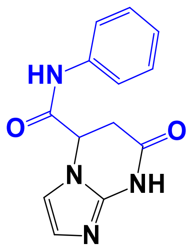

Yield 89%. White powdery compound. M.p. 253−255 ℃. ^{1}H NMR (700 MHz, DMSO-d_6) δ, ppm: 11.03 (1H, br.s, H-8), 10.42 (1H, s, CONHAr), 7.55 (2H, d, ^3J = 8.0 Hz, H_{Ar}), 7.33 (2H, t, ^3J = 7.7 Hz, H_{Ar}), 7.10 (1H, t, ^3J = 7.4 Hz, H_{Ar}), 6.96 (1H, d, ^3J = 1.6 Hz, H-3), 6.67 (1H, d, ^3J = 1.6 Hz, H-2), 5.06 (1H, dd, $^3J_{5,6A}$ = 2.8, $^3J_{5,6B}$ = 7.5 Hz, H-5), 3.25 (1H, dd, $^2J_{6A,6B}$ = 16.6, $^3J_{5,6B}$ = 7.5 Hz, H_B-6), 2.75 (1H, dd, $^2J_{6A,6B}$ = 16.6, $^3J_{5,6A}$ = 2.8 Hz, H_A-6). ^{13}C NMR (176 MHz, DMSO-d_6) δ, ppm: 167.6 (CONHAr), 166.7 (C-7), 143.3 (C-8a), 138.8 (C-1'), 129.4 (2C, C-3' and C-5'), 126.1 (C-2), 124.4 (C-4'), 119.8 (2C, C-2' and C-6'), 115.6 (C-3), 54.3 (C-5), 34.3 (C-6) ppm. HRMS (ESI) calculated for $C_{13}H_{12}N_4O_2$ [M + H]$^+$: 257.1034, found 257.1031.

N-(4-Isopropylphenyl)-7-oxo-5,6,7,8-tetrahydroimidazo[1,2-a]pyrimidine-5-carboxamide ***(4b).***

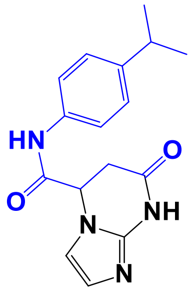

Yield 84%. White powdery compound. M.p. 271−273 ℃. ^{1}H NMR (700 MHz, DMSO-d_6) δ, ppm: 11.02 (1H, br.s, H-8), 10.35 (1H, s, CONHAr), 7.48 (2H, dd, ^4J = 2.4, ^3J = 8.0 Hz, H_{Ar}), 7.19 (1H, dd, ^4J = 2.4, ^3J = 7.8 Hz, H_{Ar}), 6.95 (1H, d, ^3J = 1.5 Hz, H-3), 6.67 (1H, d, ^3J = 1.5 Hz, H-2), 5.04 (1H, dd, $^3J_{5.6A}$ = 2.8, $^3J_{5,6B}$ = 7.5 Hz, H-5), 3.23 (1H, dd, $^2J_{6A,6B}$ = 16.6, $^3J_{5,6B}$ = 7.5 Hz, H_B-6), 2.84 (1H, hept, ^3J = 6.9 Hz, CH-*i*Pr), 2.73 (1H, dd, $^2J_{6A,6B}$ = 16.6, $^3J_{5,6A}$ = 2.8 Hz, H_A-6), 1.17 (6H, d, ^3J = 6.9 Hz, CH_3). ^{13}C NMR (176 MHz, DMSO-d_6) δ, ppm: 167.3 (CONHAr), 166.7 (C-7), 144.6 (C-1'), 143.3 (C-8a), 136.5 (C-4'), 127.1 (2C, C-3'and C-

5'), 126.1 (C-2), 119.9 (2C, C-2'and C-6'), 115.5 (C-3), 54.3 (C-5), 34.4 (C-6), 33.3 (CH-*i*Pr), 24.38 (CH_3) ppm. HRMS (ESI) calculated for $C_{16}H_{18}N_4O_2$ $[M + H]^+$: 299.1503, found 299.1503.

N-(3-Chloro-2-methylphenyl)-7-oxo-5,6,7,8-tetrahydroimidazo[1,2-a]pyrimidine-5-carboxamide ***(4c).***

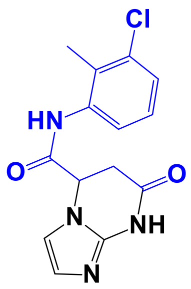

Yield 86%. White powdery compound. M.p. 278−280 ℃. ^{1}H NMR (700 MHz, DMSO-d_6) δ, ppm: 11.03 (1H, br.s, H-8), 10.05 (1H, s, CONHAr), 7.33 (1H, dd, ^3J = 8.0, ^4J = 1.3 Hz, H_{Ar}), 7.30 (1H, dd, ^3J = 8.0, ^4J = 1.3 Hz, H_{Ar}),7.22 (1H, t, ^3J = 8.0 Hz, H_{Ar}), 6.99 (1H, d, ^3J = 1.6 Hz, H-3), 6.69 (1H, d, ^3J = 1.6 Hz, H-2), 5.16 (1H, dd, $^3J_{5,6A}$ = 2.8, $^3J_{5,6B}$ = 7.5 Hz, H-5), 3.27 (1H, dd, $^2J_{6A,6B}$ = 16.6, $^3J_{5,6B}$ = 7.5 Hz, H_B-6), 2.79 (1H, dd, $^2J_{6A,6B}$ = 16.6, $^3J_{5,6A}$ = 2.8 Hz, H_A-6), 2.19 (6H, s, CH_3). ^{13}C NMR (176 MHz, DMSO-d_6) δ, ppm: 168.0 (CONHAr), 166.7 (C-7), 143.3 (C-8a), 137.3 (C-1'), 134.3 (C-3'), 131.1 (C-5'), 127.5 (C-4'), 127.2 (C-2'), 126.2 (C-2), 125.0 (C-6'), 115.5 (C-3), 54.8 (C-5), 34.4 (C-6), 15.4 (CH_3) ppm. HRMS (ESI) calculated for $C_{14}H_{13}ClN_4O_2$ $[M + H]^+$: 305.0800, found 305.0800.

N-(2,5-Dichlorophenyl)-7-oxo-5,6,7,8-tetrahydroimidazo[1,2-a]pyrimidine-5-carboxamide ***(4d)****.*

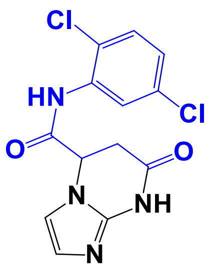

Yield 83%. White powdery compound. M.p. 248−250 ℃. ^{1}H NMR (700 MHz, DMSO-d_6) δ, ppm: 10.98 (1H, br.s, H-8), 10.09 (1H, s, CONHAr), 7.73 (1H, d, ^4J = 2.4 Hz, H_{Ar}), 7.49 (1H, d, ^3J = 8.6 Hz, H_{Ar}), 7.23 (1H, dd, ^4J = 2.4, ^3J = 8.6 Hz, H_{Ar}), 6.90 (1H, d, ^3J = 1.1 Hz, H-3), 6.60 (1H, d, ^3J = 1.1 Hz, H-2), 5.19 (1H, dd, $^3J_{5,6A}$ = 2.6, $^3J_{5,6B}$ = 7.5 Hz, H-5), 3.17 (1H, dd, $^2J_{6A,6B}$ = 16.6, $^3J_{5,6B}$ = 7.5 Hz, H_B-6), 2.72 (1H, dd, $^2J_{6A,6B}$ = 16.6, $^3J_{5,6A}$ = 2.8 Hz, H_A-6). ^{13}C NMR (176 MHz, DMSO-d_6) δ, ppm:

168.5 (CONHAr), 166.6 (C-7), 143.3 (C-8a), 135.7 (C-1’), 132.1 (C-5’), 131.5 (C-3’), 127.1 (C-4’), 126.2 (C-2), 125.7 (C-6’), 125.6 (C-2’), 115.6 (C-3), 53.7 (C-5), 34.3 (C-6) ppm. HRMS (ESI) calculated for $C_{13}H_{10}Cl_2N_4O_2$ $[M + H]^+$: 325.0254, found 325.0254.

N-(2-Methyl-5-nitrophenyl)-7-oxo-5,6,7,8-tetrahydroimidazo[1,2-a]pyrimidine-5-carboxamide ***(4e).***

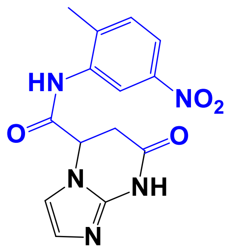

Yield 82%. Pale yellow powdery compound. M.p. 275−277℃. ^{1}H NMR (700 MHz, DMSO-d_6) δ, ppm: 11.09 (1H, br.s, H-8), 10.08 (1H, s, CONHAr), 8.40 (1H, d, ^4J = 2.5 Hz, H_{Ar}), 7.98 (1H, dd, ^3J = 8.5, ^4J = 2.6 Hz, H_{Ar}), 7.53 (1H, d, ^3J = 8.5 Hz, H_{Ar}), 7.01 (1H, d, ^3J = 1.5 Hz, H-3), 6.70 (1H, d, ^3J = 1.5 Hz, H-2), 5.25 (1H, dd, $^3J_{5,6A}$ = 2.8, $^3J_{5,6B}$ = 7.5 Hz, H-5), 3.30 (1H, dd, $^2J_{6A,6B}$ = 16.6, $^3J_{5,6B}$ = 7.5 Hz, H_B-6), 2.84 (1H, dd, $^2J_{6A,6B}$ = 16.6, $^3J_{5,6A}$ = 2.6 Hz, H_A-6), 2.35 (3H, s, CH_3). ^{13}C NMR (176 MHz, DMSO-d_6) δ, ppm: 168.5 (CONHAr), 166.7 (C-7), 146.1 (C-5’), 143.3 (C-8a), 140.0 (C-1’), 136.8 (C-2’), 132.1 (C-3’), 126.2 (C-2), 120.5 (C-4’), 119.1 (C-6’), 115.6 (C-3), 53.9 (C-5), 34.3 (C-6), 18.5 (CH_3) ppm. HRMS (ESI) calculated for $C_{14}H_{13}N_5O_4$ $[M + H]^+$: 316.1041, found 316.1041.

*N-Benzyl-7-oxo-5,6,7,8-tetrahydroimidazo[1,2-a]pyrimidine-5-carboxamide (**4f**).*

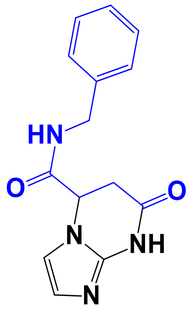

Yield 84%. Pale pink powdery compound. M.p. 237−239 ℃. ^{1}H NMR (700 MHz, DMSO-d_6) δ, ppm: 10.99 (1H, s, H-8), 8.82 (1H, t, ^3J = 5.8 Hz, CONHAlk), 7.33 (2H, t, ^3J = 7.6 Hz, H_{Ar}), 7.28–7.22 (3H, m, H_{Ar}), 6.88 (1H, d, ^3J = 1.5 Hz, H-3), 6.66 (1H, d, ^3J = 1.5 Hz, H-2), 4.93 (1H, dd, $^3J_{5,6A}$ = 2.9, $^3J_{5,6B}$ = 7.4 Hz, H-5), 4.30 (2H, d, ^3J = 5.6 Hz, H-1’), 3.14 (1H, dd, $^2J_{6A,6B}$ = 16.5, $^3J_{5,6B}$ = 7.4 Hz, H_B-6), 2.66 (1H, dd, $^2J_{6A,6B}$ =

16.5, $^3J_{5,6A}$ = 2.9 Hz, H_A-6). ^{13}C NMR (176 MHz, DMSO-d_6) δ, ppm: 168.9 (CONHAlk), 166.8 (C-7), 143.2 (C-8a), 139.1 (C-1''), 128.9 (2C, C-3'' and C-5''), 127.7 (2C, C-2'' and C-6''), 127.5 (C-4''), 126.0 (C-2), 115.4 (C-3), 53.6 (C-5), 42.8 (C-1'), 34.3 (C-6). HRMS (ESI) calculated for $C_{14}H_{14}N_4O_2$ [M + H]$^+$: 271.1190, found 271.1191.

*7-Oxo-N-phenethyl-5,6,7,8-tetrahydroimidazo[1,2-a]pyrimidine-5-carboxamide (**4g**).*

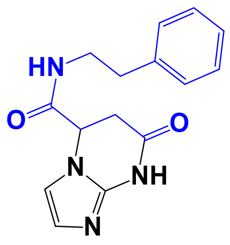

Yield 75%. Pale pink powdery compound. M.p. 232−234 ℃. ^{1}H NMR (700 MHz, DMSO-d_6) δ, ppm: 10.96 (1H, s, H-8), 8.38 (1H, t, ^3J = 5.6 Hz, CONHAlk), 7.30 (2H, t, ^3J = 7.6 Hz, H_{Ar}), 7.24–7.14 (3H, m, H_{Ar}), 6.75 (1H, d, ^3J = 1.6 Hz, H-3), 6.64 (1H, d, ^3J = 1.6 Hz, H-2), 4.80 (1H, dd, $^3J_{5,6A}$ = 3.3, $^3J_{5,6B}$ = 7.4 Hz, H-5), 3.39–3.23 (2H, m, H-2'), 3.05 (1H, dd, $^2J_{6A,6B}$ = 16.5, $^3J_{5,6B}$ = 7.4 Hz, H_B-6), 2.71 (2H, t, ^3J = 7.2 Hz, H-1'), 2.56 (1H, dd, $^2J_{6A,6B}$ = 16.5, $^3J_{5,6A}$ = 3.3 Hz, H_A-6). ^{13}C NMR (176 MHz, DMSO-d_6) δ, ppm: 168.7 (CONHAlk), 166.8 (C-7), 143.1 (C-8a), 139.6 (C-1''), 129.2 (2C, C-3'' and C-5''), 128.8 (2C, C-2'' and C-6''), 126.7 (C-4''), 125.9 (C-2), 115.3 (C-3), 53.5 (C-5), 40.8 (C-1'), 35.2 (C-2'), 34.3 (C-6). HRMS (ESI) calculated for $C_{15}H_{16}N_4O_2$ [M + H]$^+$: 285.1347, found 285.1344.

*N-(4-Methoxyphenethyl)-7-oxo-5,6,7,8-tetrahydroimidazo[1,2-a]pyrimidine-5-carboxamide (**4h**).*

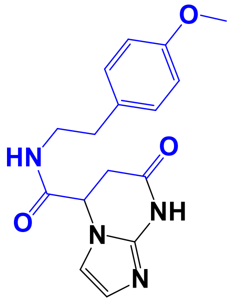

Yield 89%. Pale pink powdery compound. M.p. 249−251 ℃. ^{1}H NMR (700 MHz, DMSO-d_6) δ, ppm: 10.95 (1H, s, H-8), 8.34 (1H, t, ^3J = 5.7 Hz, CONHAlk), 7.11 – 7.09 (2H, m, H_{Ar}), 6.87–6.84 (2H, m, H_{Ar}), 6.75 (1H, d, ^3J = 1.5 Hz, H-3), 6.64 (1H, d, ^3J = 1.5 Hz, H-2), 4.80 (1H, dd, $^3J_{5,6A}$ = 3.2, $^3J_{5,6B}$ = 7.4 Hz, H-5), 3.73 (3H, s, OCH_3), 3.30–3.21 (2H, m,

H-2'), 3.06 (1H, dd, $^2J_{6A,6B}$ = 16.5, $^3J_{5,6B}$ = 7.4 Hz, H_B-6), 2.64 (2H, t, ^3J = 7.1 Hz, H-1'), 2.56 (1H, dd, $^2J_{6A,6B}$ = 16.5, $^3J_{5,6A}$ = 3.2 Hz, H_A-6). ^{13}C NMR (176 MHz, DMSO-d_6) δ, ppm: 168.6 (CONHAlk), 166.8 (C-7), 158.2 (C-4''), 143.1 (C-8a), 131.4 (C-1''), 130.1 (2C, C-2'' and C-6''), 125.9 (C-2), 115.4 (C-3), 114.3 (2C, C-3'' and C-5''), 55.5 (CH_3), 53.5 (C-5), 41.0 (C-1'), 34.4 (C-2''), 34.3 (C-6). HRMS (ESI) calculated for $C_{16}H_{18}N_4O_3$ [M + H]$^+$: 315.1453, found 315.1451.

*N-Methyl-7-oxo-5,6,7,8-tetrahydroimidazo[1,2-a]pyrimidine-5-carboxamide (**4i**).*

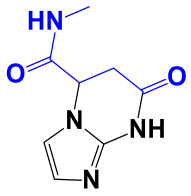

Yield 70%. Pale pink powdery compound. M.p. 218−220 ℃. ^{1}H NMR (700 MHz, DMSO-d_6) δ, ppm: 10.95 (1H, s, H-8), 8.41 (1H, t, ^3J = 4.6 Hz, CONHAlk), 6.82 (1H, d, ^3J = 1.6 Hz, H-3), 6.62 (1H, d, ^3J = 1.6 Hz, H-2), 4.85 (1H, dd, $^3J_{5,6A}$ = 3.1, $^3J_{5,6B}$ = 7.4 Hz, H-5), 3.04 (1H, dd, $^2J_{6A,6B}$ = 16.8, $^3J_{5,6B}$ = 7.8 Hz, H_B-6), 2.63–2.55 (4H, m, CH_3 and H_A-6). ^{13}C NMR (176 MHz, DMSO-d_6) δ, ppm: 169.3 ($CONHCH_3$), 167.1 (C-7), 143.5 (C-8a), 125.9 (C-2), 115.3 (C-3), 53.5 (C-5), 34.3 (C-6), 26.1 (CH_3). HRMS (ESI) calculated for $C_8H_{10}N_4O_2$ [M + H]$^+$: 195.0877, found 195.0878.

*N-Phenyl-2-(7-oxo-5,6,7,8-tetrahydroimidazo[1,2-a]pyrimidin-6-yl)acetamide (**5a**).*

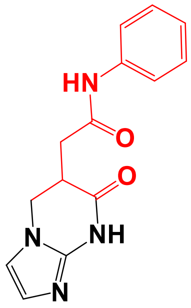

Yield 92%. White powdery compound. M.p. 242−244 ℃. ^{1}H NMR (700 MHz, DMSO-d_6) δ, ppm: 11.09 (1H, br.s, H-8), 10.07 (1H, s, CONHAr), 7.59 (2H, d, ^3J = 7.6 Hz, H_{Ar}), 7.30 (2H, t, ^3J = 7.7 Hz, H_{Ar}), 7.04 (1H, t, ^3J = 7.4 Hz, H_{Ar}), 6.91 (1H, d, ^3J = 1.4 Hz, H-3), 6.65 (1H, d, ^3J = 1.2 Hz, H-2), 4.28 (1H, dd, $^3J_{5A,6}$ = 6.9, $^2J_{5A,5B}$ = 12.4 Hz, H_A-5), 3.92 (1H, t, $^3J_{5B,6}$ = $^2J_{5A,5B}$ = 12.3 Hz, H_B-5), 3.19−3.26 (1H, m, H-6), 2.91 (1H, dd,

$^{2}J_{2'A,2'B}$ = 16.2, $^{3}J_{2'A,6}$ = 4.5 Hz, H$_A$-2'), 2.55 (1H, dd, $^{2}J_{2'A,2'B}$ = 16.2, $^{3}J_{2'B,6}$ = 7.9 Hz, H$_B$-2'). ^{13}C NMR (176 MHz, DMSO-*d*$_6$) δ, ppm: 169.5 (<u>C</u>ONHAr), 169.4 (C-7), 142.6 (C-8a), 139.6 (C-1'), 129.1 (2C, C-3' and C-5'), 125.5 (C-2), 123.6 (C-4'), 119.5 (2C, C-2' and C-6'), 116.0 (C-3), 44.1 (C-5), 37.0 (C-6), 34.5 (C-2'') ppm. HRMS (ESI) calculated for $C_{14}H_{14}N_4O_2$ [M + H]$^+$: 271.1190, found 271.1191.

N-(4-Bromophenyl)-2-(7-oxo-5,6,7,8-tetrahydroimidazo[1,2-a]pyrimidin-6-yl)acetamide ***(5b)***.

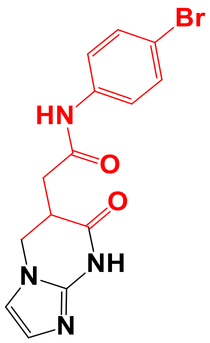

Yield 86%. Pale yellow powdery compound. M.p. 263−265 ℃. ^{1}H NMR (700 MHz, DMSO-*d*$_6$) δ, ppm: 11.07 (1H, br.s, H-8), 10.21 (1H, s, CONHAr), 7.57 (2H, dd, ^{3}J = 8.9, ^{4}J = 0.8, H$_{Ar}$), 7.48 (2H, dd, ^{3}J = 8.9, ^{4}J = 1.1, H$_{Ar}$), 6.90 (1H, d, ^{3}J = 1.5 Hz, H-3), 6.65 (1H, d, ^{3}J = 1.5 Hz, H-2), 4.28 (1H, dd, $^{3}J_{5A,6}$ = 7.6, $^{2}J_{5A,5B}$ = 12.5 Hz, H$_A$-5), 3.92 (1H, t, $^{3}J_{5B,6}$= $^{2}J_{5A,5B}$ = 12.1 Hz, H$_B$-5), 3.19−3.25 (1H, m, H-6), 2.91 (1H, dd, $^{2}J_{2'A,2'B}$ = 16.1, $^{3}J_{2'A,6}$ = 4.8 Hz, H$_A$-2'), 2.55 (1H, dd, $^{2}J_{2'A,2'B}$ = 16.1, $^{3}J_{2'B,6}$ = 7.7 Hz, H$_B$-2'). ^{13}C NMR (176 MHz, DMSO-*d*$_6$) δ, ppm: 169.7 (<u>C</u>ONHAr), 169.4 (C-7), 142.6 (C-8a), 139.0 (C-1'), 132.0 (2C, C-3' and C-5'), 125.5 (C-2), 121.4 (2C, C-2' and C-6'), 116.0 (C-3), 115.1 (C-4'), 44.0 (C-5), 37.9 (C-6), 34.5 (C-2'') ppm. HRMS (ESI) calculated for $C_{14}H_{13}BrN_4O_2$ [M + H]$^+$: 349.0295, found 349.0294.

N-(4-Fluorophenyl)-2-(7-oxo-5,6,7,8-tetrahydroimidazo[1,2-a]pyrimidin-6-yl)acetamide ***(5c)***.

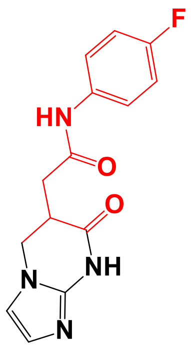

Yield 83%. White powdery compound. M.p. 238−240 ℃. ^{1}H NMR (700 MHz, DMSO-*d*$_6$) δ, ppm: 11.07 (1H, br.s, H-8), 10.13 (1H, s, CONHAr), 7.58−7.64 (2H, m, H$_{Ar}$), 7.14 (2H, td, ^{3}J = 9.0, ^{4}J = 2.1, H$_{Ar}$), 6.90 (1H, d, ^{3}J = 1.5 Hz, H-3), 6.65 (1H, d, ^{3}J = 1.5 Hz, H-2), 4.28 (1H, dd, $^{3}J_{5A,6}$ =

7.0, $^{2}J_{5A,5B}$ = 12.4 Hz, H_A-5), 3.92 (1H, t, $^{3}J_{5B,6}$ = $^{2}J_{5A,5B}$ = 12.1 Hz, H_B-5), 3.19−3.25 (1H, m, H-6), 2.91 (1H, dd, $^{2}J_{2'A,2'B}$ = 16.0, $^{3}J_{2'A,6}$ = 4.7 Hz, H_A-2'), 2.55 (1H, dd, $^{2}J_{2'A,2'B}$ = 16.1, $^{3}J_{2'B,6}$ = 7.8 Hz, H_B-2'). ^{13}C NMR (176 MHz, DMSO-d_6) δ, ppm: 169.5 (<u>C</u>ONHAr), 169.3 (C-7), 159.0 and 157.7 (C-F), 142.6 (C-8a), 136.0 (C-1'), 125.5 (C-2), 121.2 (2C, C-2' and C-6'), 116.0 (C-3), 115.8 and 115.7 (C-3' and C-5'), 44.0 (C-5), 37.0 (C-6), 34.4 (C-2'') ppm. ^{19}F NMR (659 MHz, DMSO-d_6) δ, ppm: −119.55. HRMS (ESI) calculated for $C_{14}H_{13}FN_4O_2$ [M + H]$^+$: 289.1096, found 289.1096.

N-(4-Chlorophenyl)-2-(7-oxo-5,6,7,8-tetrahydroimidazo[1,2-a]pyrimidin-6-yl)acetamide ***(5d)***.

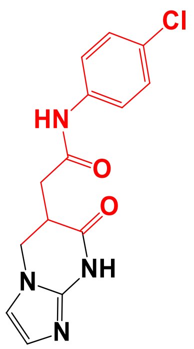

Yield 87%. White powdery compound. M.p. 239−241 ℃. ^{1}H NMR (700 MHz, DMSO-d_6) δ, ppm: 11.09 (1H, br.s, H-8), 10.42 (1H, s, CONHAr), 7.64 (2H, dt, ^{3}J = 8.8, ^{4}J = 3.1 Hz, H_{Ar}), 7.36 (2H, dt, ^{3}J = 8.8, ^{4}J = 1.9 Hz, H_{Ar}), 6.90 (1H, d, ^{3}J = 1.4 Hz, H-3), 6.65 (1H, d, ^{3}J = 1.4 Hz, H-2), 4.27 (1H, dd, $^{3}J_{5A,6}$ = 7.2, $^{2}J_{5A,5B}$ = 12.3 Hz, H_A-5), 3.91 (1H, t, $^{3}J_{5B,6}$ = $^{2}J_{5A,5B}$ = 12.3 Hz, H_B-5), 3.19−3.25 (1H, m, H-6), 2.91 (1H, dd, $^{2}J_{2'A,2'B}$ = 16.2, $^{3}J_{2'A,6}$ = 4.8 Hz, H_A-2'), 2.56 (1H, dd, $^{2}J_{2'A,2'B}$ = 16.2, $^{3}J_{2'B,6}$ = 7.9 Hz, H_B-2'). ^{13}C NMR (176 MHz, DMSO-d_6) δ, ppm: 169.7 (<u>C</u>ONHAr), 169.5 (C-7), 142.6 (C-8a), 138.6 (C-1'), 129.0 (2C, C-3' and C-5'), 127.0 (C-4'), 125.5 (C-2), 121.1 (2C, C-2' and C-6'), 116.0 (C-3), 44.0 (C-5), 37.0 (C-6), 34.5 (C-2'') ppm. HRMS (ESI) calculated for $C_{14}H_{13}ClN_4O_2$ [M + H]$^+$: 305.0800, found 305.0801.

N-(4-Ethylphenyl)-2-(7-oxo-5,6,7,8-tetrahydroimidazo[1,2-a]pyrimidin-6-yl)acetamide ***(5e)***.

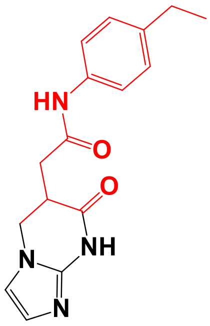

Yield 88%. White powdery compound. M.p. 215−217 ℃. ^{1}H NMR (700 MHz, DMSO-d_6) δ, ppm: 11.11 (1H, br.s, H-8), 10.09 (1H, s, CONHAr), 7.50 (2H, dd, ^4J = 1.2 Hz, ^3J = 8.6 Hz, H_{Ar}), 7.12 (2H, dd, ^4J = 1.2 Hz, ^3J = 8.6 Hz, H_{Ar}), 6.90 (1H, d, ^3J = 1.4 Hz, H-3), 6.65 (1H, d, ^3J = 1.1 Hz, H-2), 4.27 (1H, dd, $^3J_{5A,6}$ = 6.9, $^2J_{5A,5B}$ = 12.4 Hz, H_A-5), 3.91 (1H, t, $^3J_{5B,6}$ = $^2J_{5A,5B}$ = 12.2 Hz, H_B-5), 3.19−3.24 (1H, m, H-6), 2.91 (1H, dd, $^2J_{2'A,2'B}$ = 16.0, $^3J_{2'A,6}$ = 4.5 Hz, H_A-2'), 2.52−2.57 (3H, m, H_B-2' and $\underline{CH_2}CH_3$), 1.15 (3H, t, ^3J = 7.6 Hz, $CH_2\underline{CH_3}$). ^{13}C NMR (176 MHz, DMSO-d_6) δ, ppm: 169.5 (<u>C</u>ONHAr), 169.2 (C-7), 142.7 (C-8a), 138.9 (C-4'), 137.4 (C-1'), 128.3 (2C, C-3' and C-5'), 125.5 (C-2), 119.6 (2C, C-2' and C-6'), 116.0 (C-3), 44.1 (C-5), 37.0 (C-6), 34.4 (C-2''), 28.1 (CH_2), 16.2 (CH_3) ppm. HRMS (ESI) calculated for $C_{16}H_{18}N_4O_2$ [M + H]$^+$: 299.1507, found 299.1509.

3. NMR and mass spectra of products

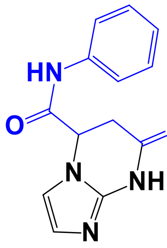

4a

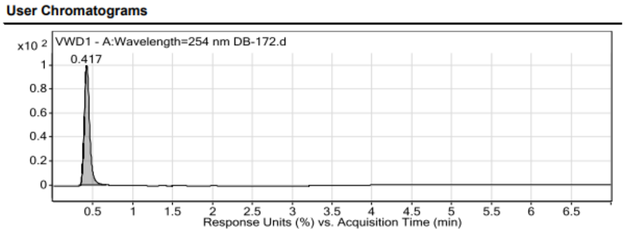

Integration Peak List

Peak	Start	RT	End	Height	Area	Area %
1	0.32	0.417	0.66	758.87	3600.09	100

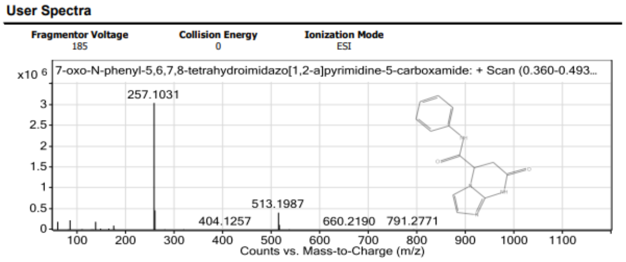

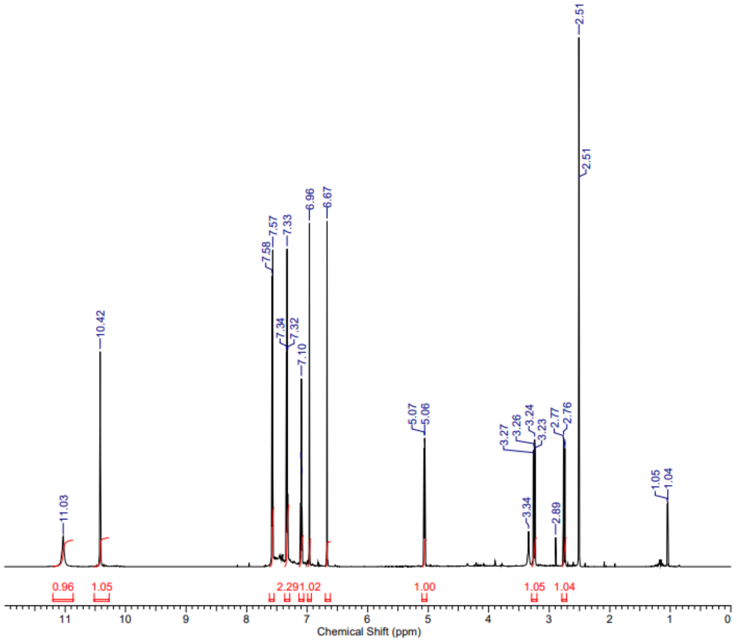

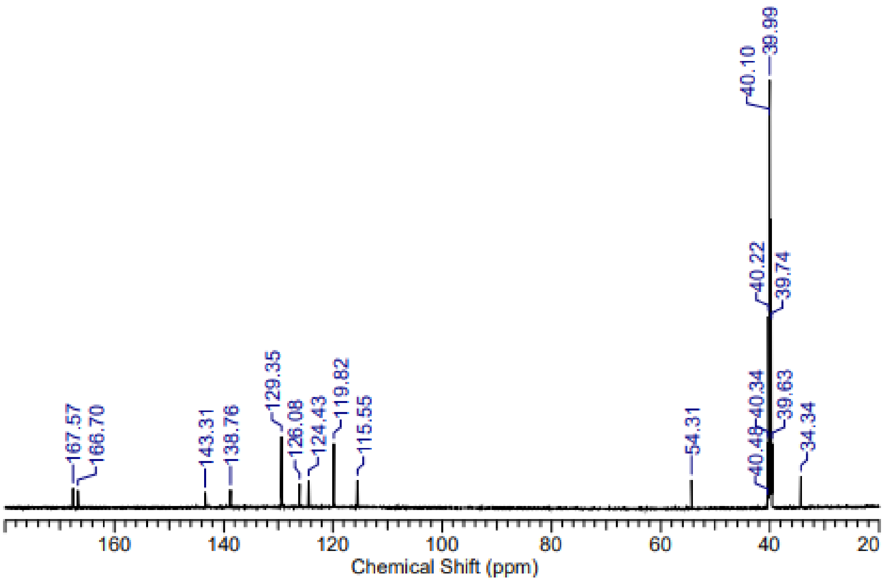

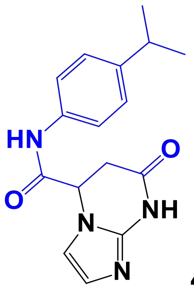

4b

User Chromatograms

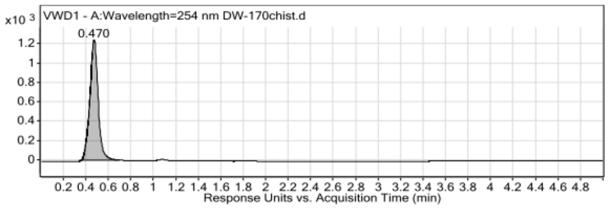

Integration Peak List

Peak	Start	RT	End	Height	Area	Area %
1	0.34	0.47	0.693	1246.58	6590.52	100

User Spectra

Fragmentor Voltage	Collision Energy	Ionization Mode
185	0	ESI

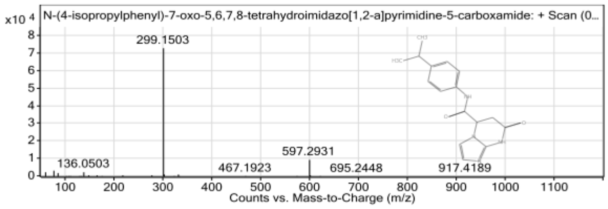

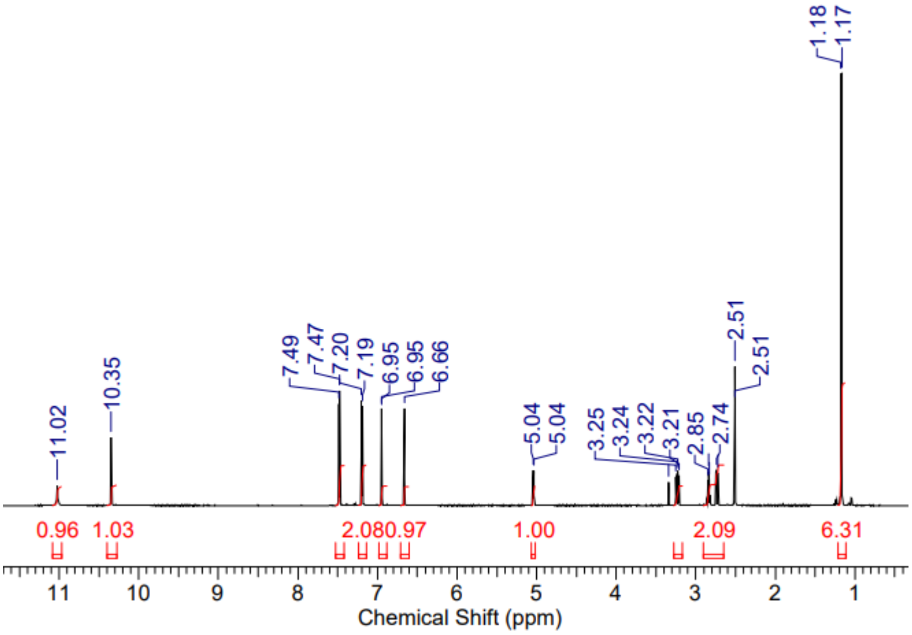

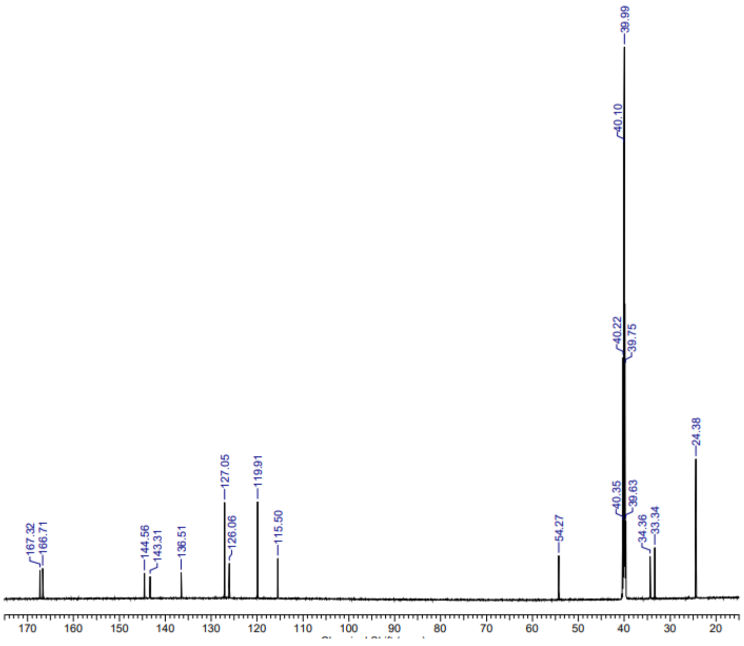

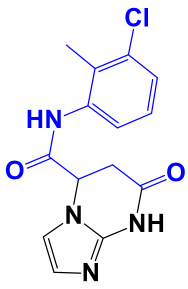

4c

User Chromatograms

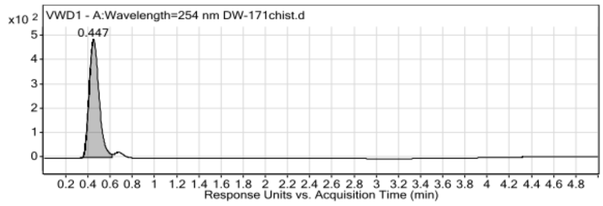

Integration Peak List

Peak	Start	RT	End	Height	Area	Area %
1	0.326	0.447	0.617	487.57	3046.41	100

User Spectra

Fragmentor Voltage	Collision Energy	Ionization Mode
185	0	ESI

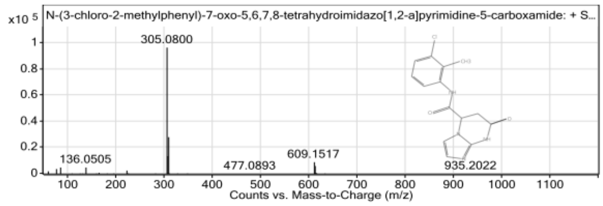

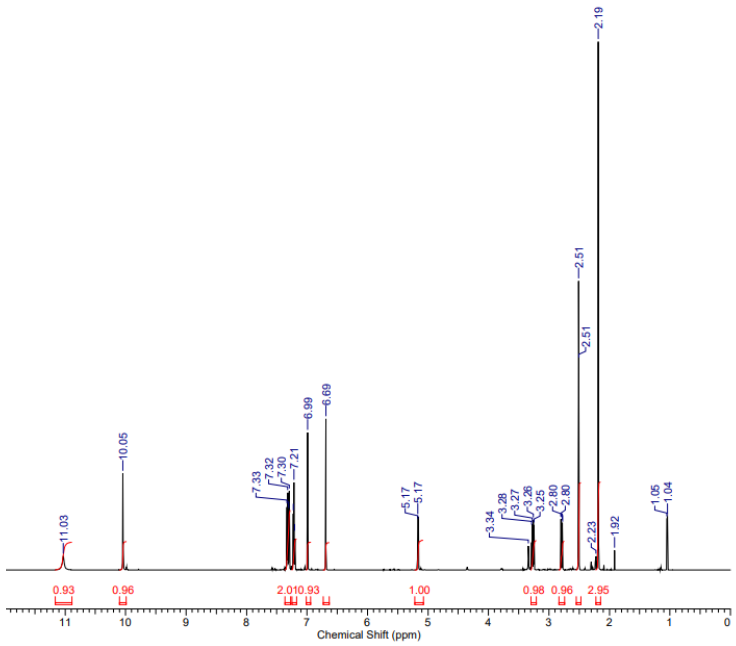

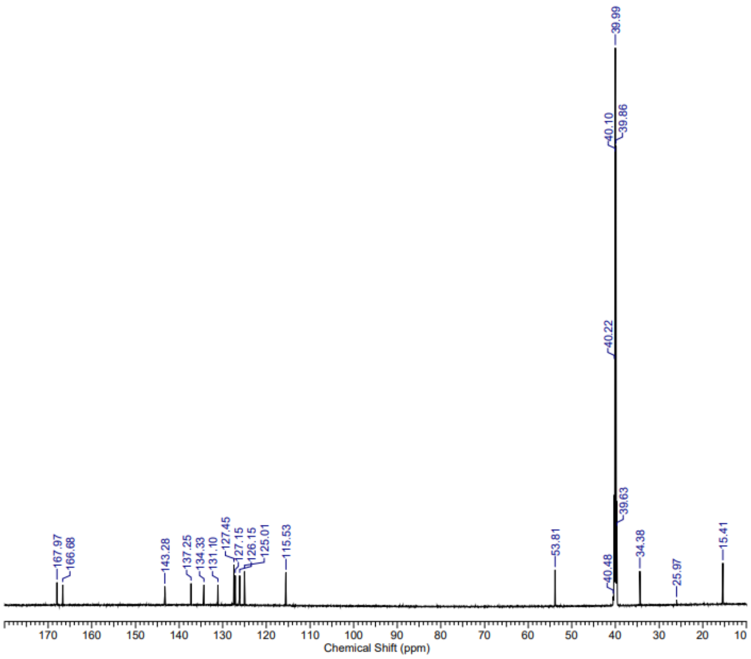

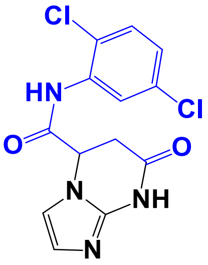

4d

User Chromatograms

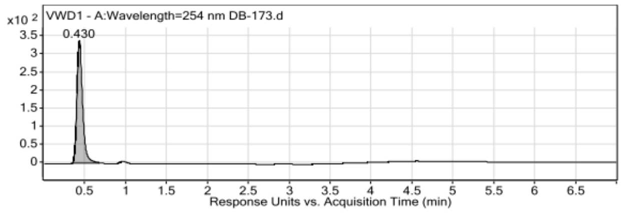

Integration Peak List

Peak	Start	RT	End	Height	Area	Area %
1	0.331	0.43	0.673	340.36	1692.22	100

User Spectra

Fragmentor Voltage	Collision Energy	Ionization Mode
185	0	ESI

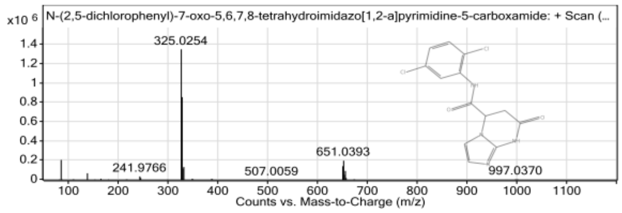

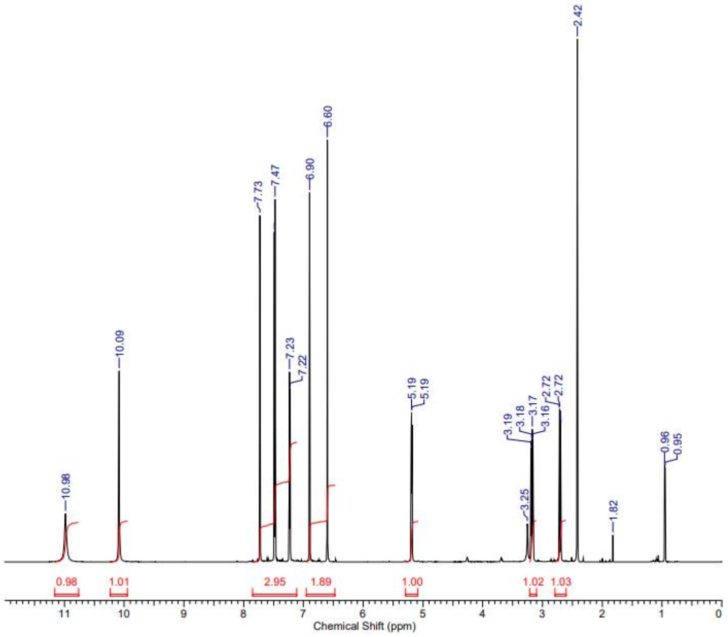

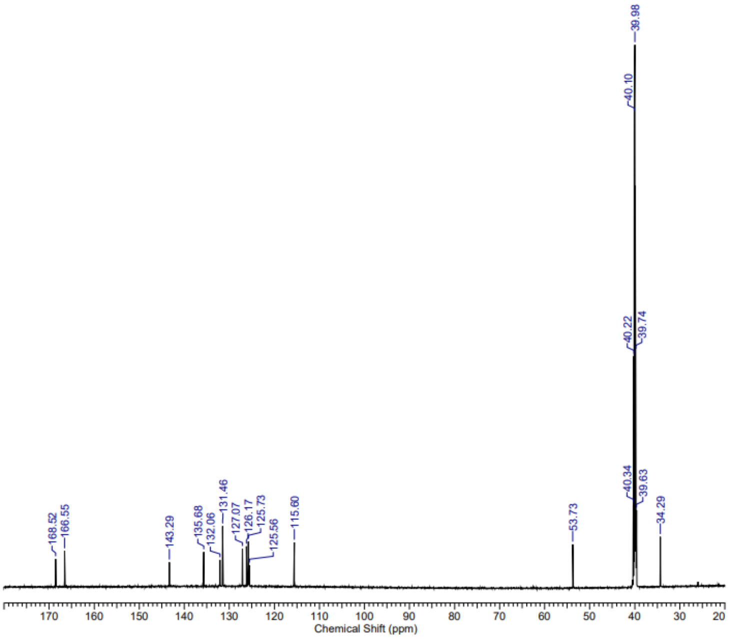

FZ2918.028.001.2rr NOESY-1.esp

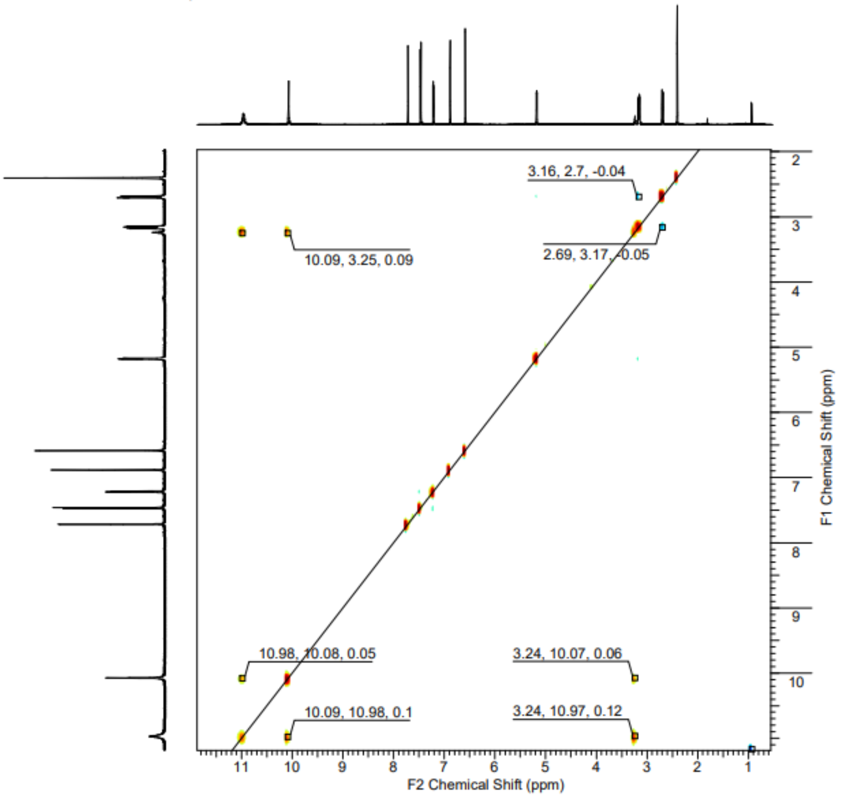

FZ2918.026.001.2rr HMBC.esp

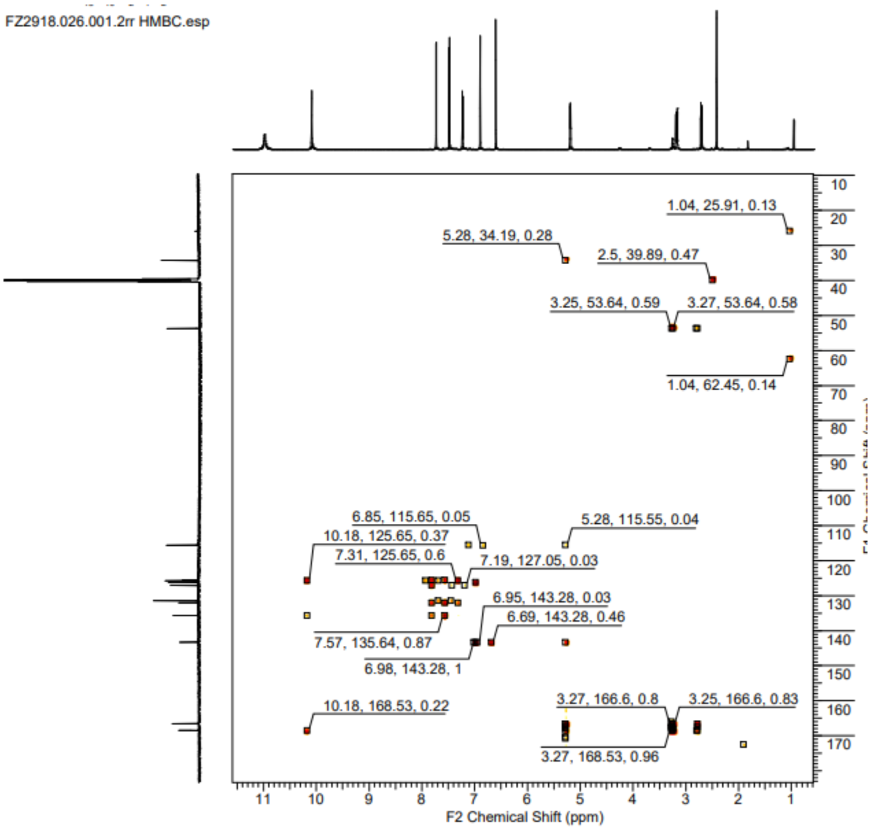

User Chromatograms

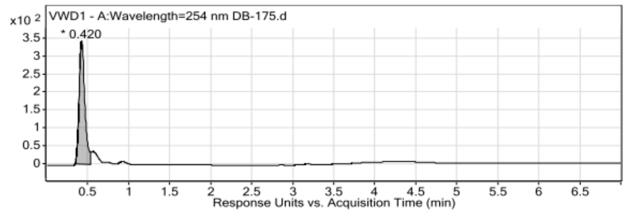

Integration Peak List

Peak	Start	RT	End	Height	Area	Area %
1	0.334	0.42	0.537	345.6	1711.22	100

User Spectra

Fragmentor Voltage	Collision Energy	Ionization Mode
185	0	ESI

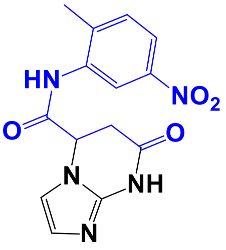

4e

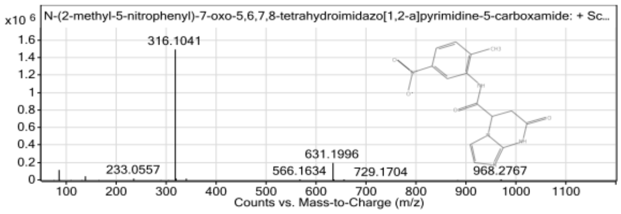

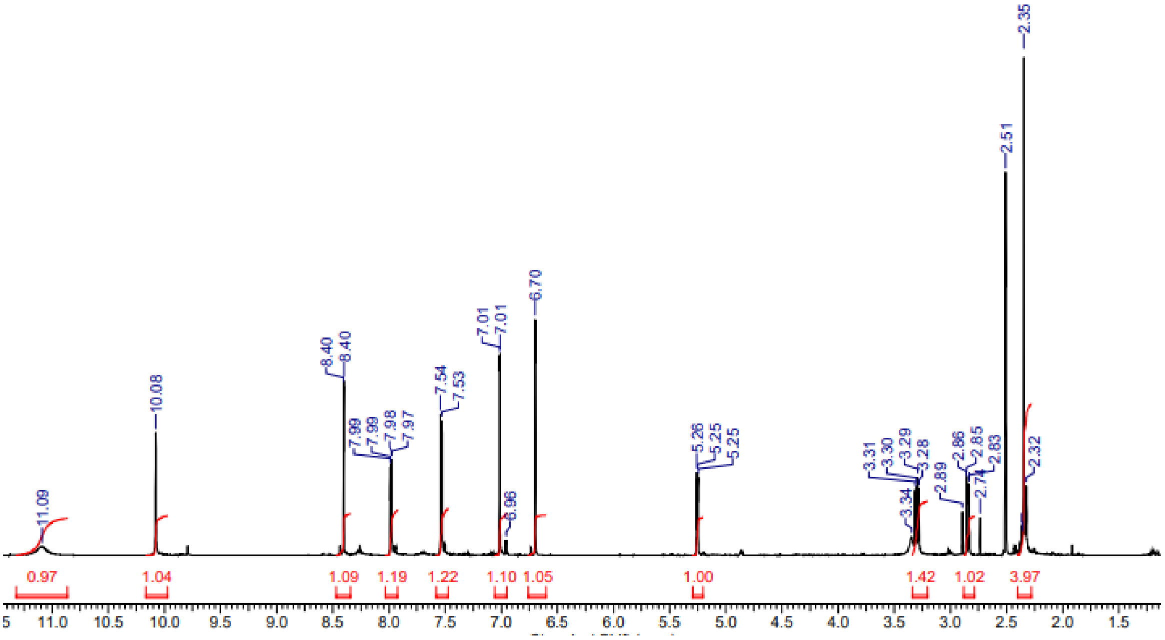

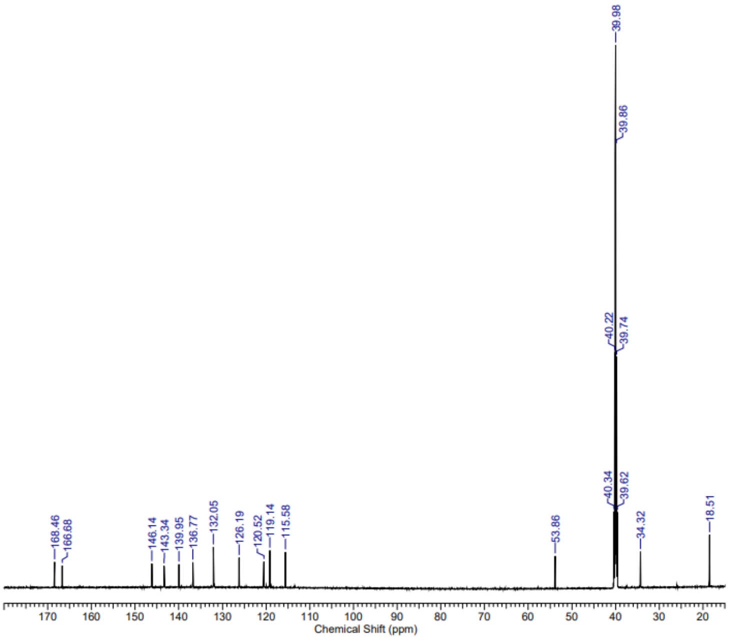

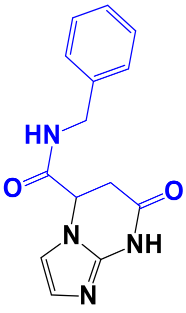

4f

User Chromatograms

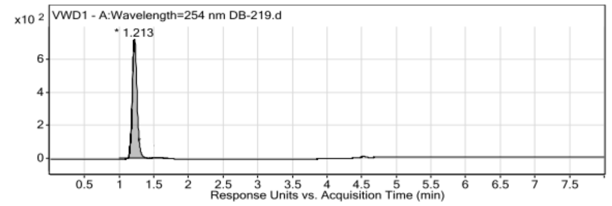

Integration Peak List

Peak	Start	RT	End	Height	Area	Area %
1	0.997	1.213	1.443	725.62	3509.57	100

User Spectra

Fragmentor Voltage	Collision Energy	Ionization Mode
185	0	ESI

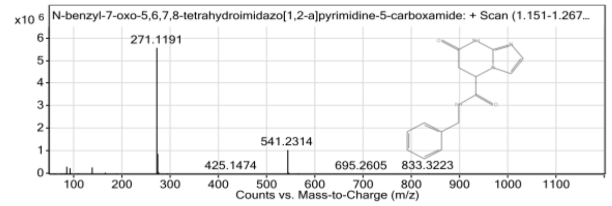

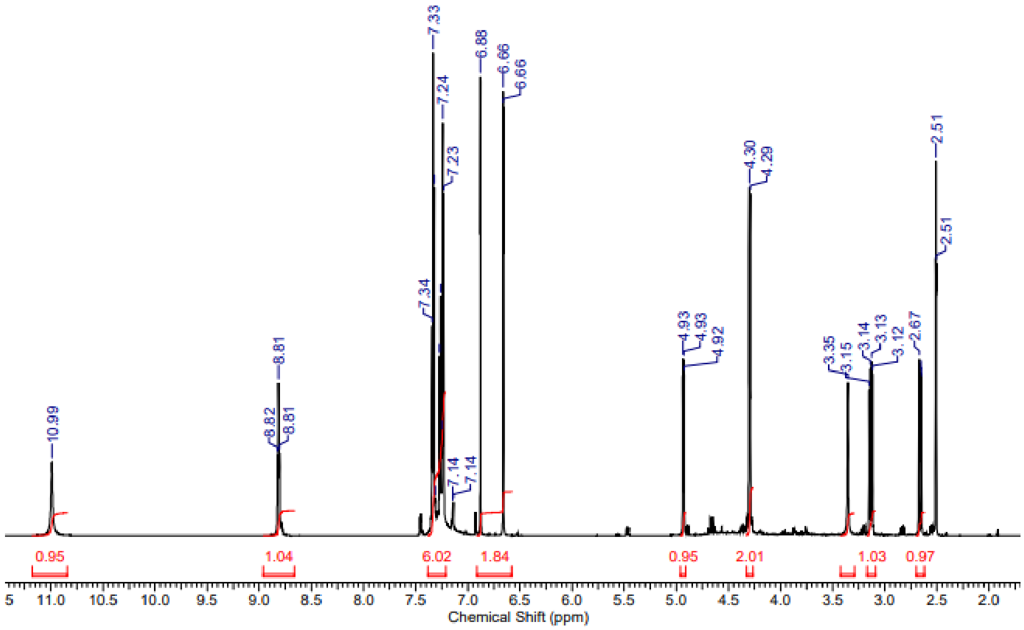

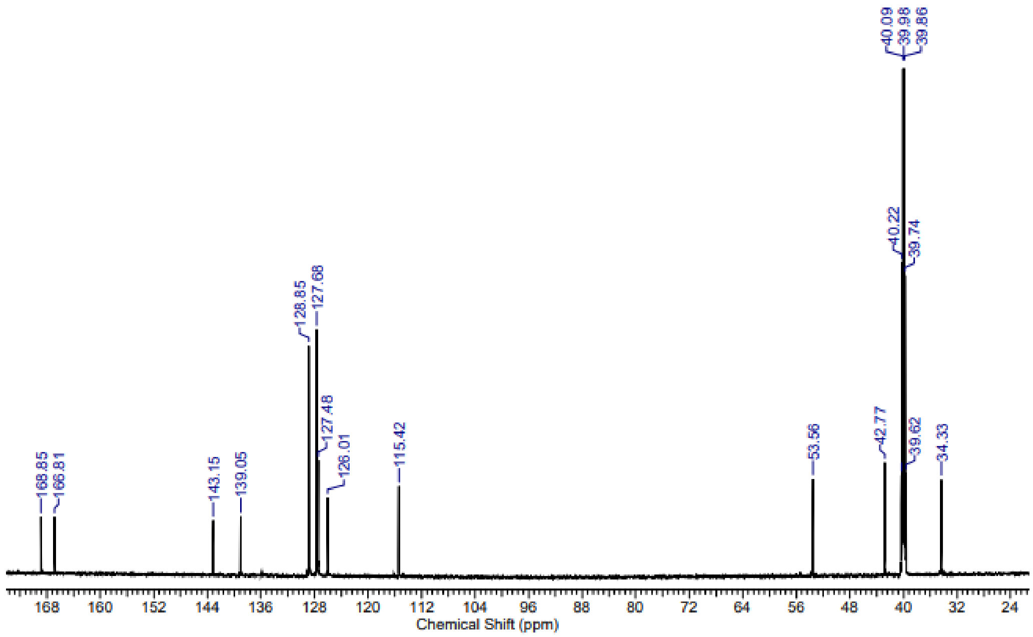
168.85
166.81
143.15
139.05
128.85
127.68
127.48
126.01
115.42
53.56
42.77
40.22
40.09
39.98
39.86
39.74
39.62
34.33
168
160
152
144
136
128
120
112
104
96
88
80
72
64
56
48
40
32
24
Chemical Shift (ppm)

User Chromatograms

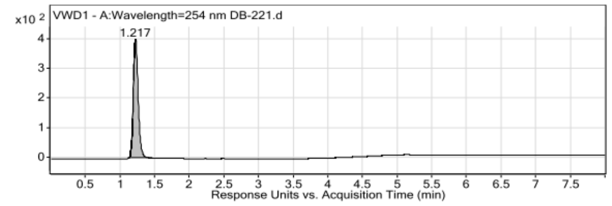

Integration Peak List

Peak	Start	RT	End	Height	Area	Area %
1	1.113	1.217	1.46	400.74	1968.15	100

User Spectra

Fragmentor Voltage	Collision Energy	Ionization Mode
185	0	ESI

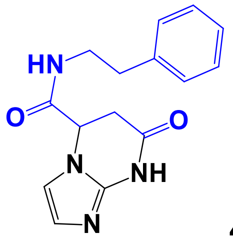

4g

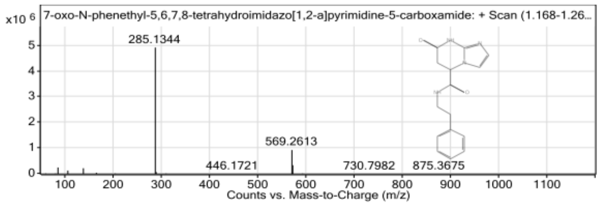

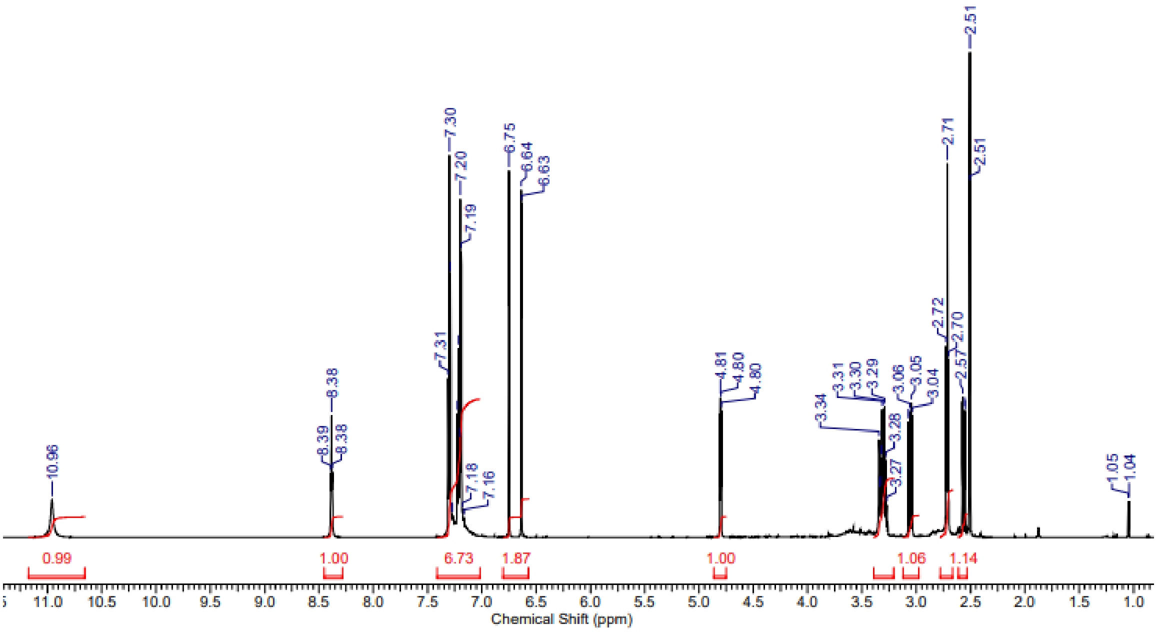

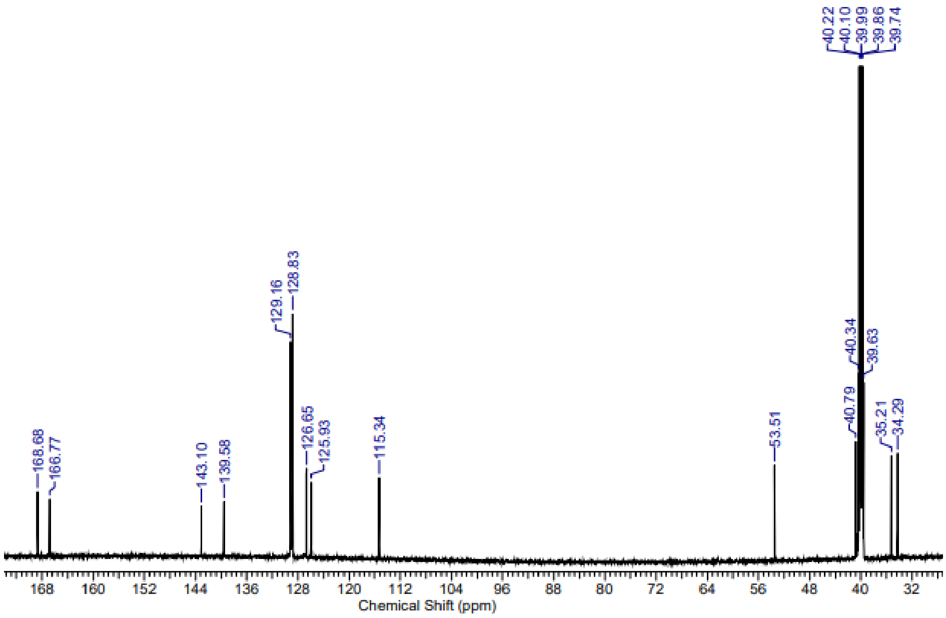

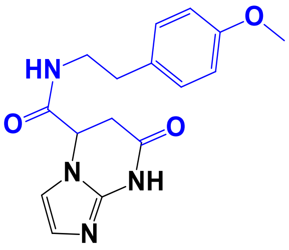

4h

User Chromatograms

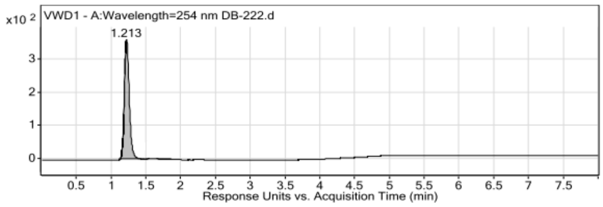

Integration Peak List

Peak	Start	RT	End	Height	Area	Area %
1	1.112	1.213	1.477	359.67	1787.12	100

User Spectra

Fragmentor Voltage	Collision Energy	Ionization Mode
185	0	ESI

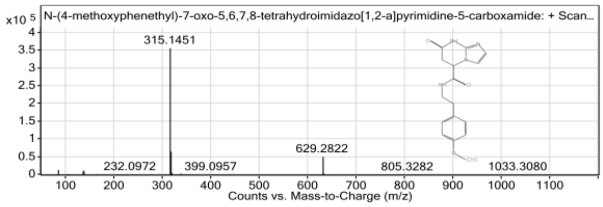

FZ3548_010001R.ESP

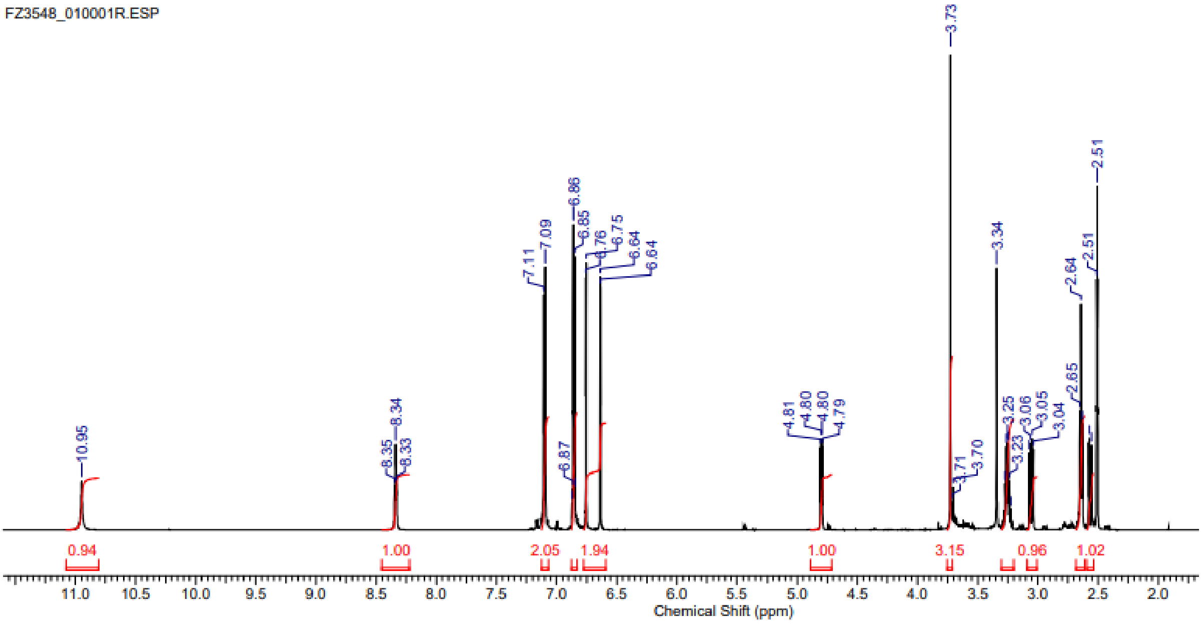

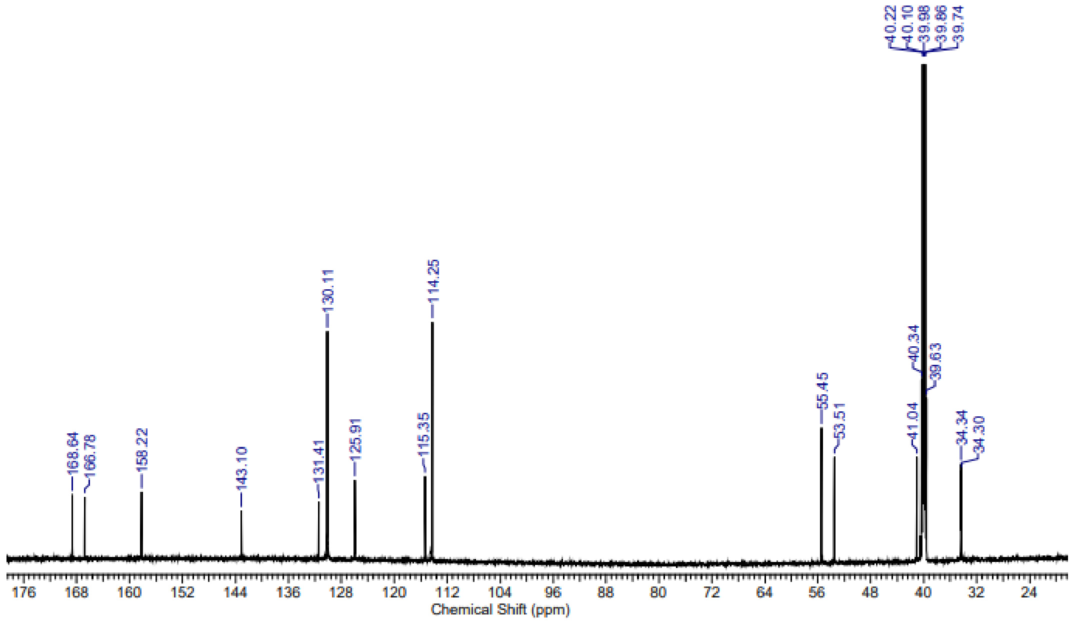

168.64
166.78
158.22
143.10
131.41
130.11
125.91
115.35
114.25
55.45
53.51
41.04
40.34
40.22
40.10
39.98
39.86
39.74
39.63
34.34
34.30
176
168
160
152
144
136
128
120
112
104
96
88
80
72
64
56
48
40
32
24
Chemical Shift (ppm)

User Chromatograms

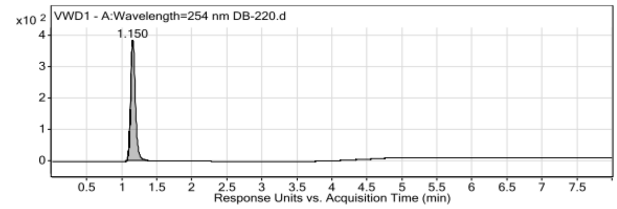

Integration Peak List

Peak	Start	RT	End	Height	Area	Area %
1	1.05	1.15	1.373	386.15	1809.65	100

User Spectra

Fragmentor Voltage	Collision Energy	Ionization Mode
185	0	ESI

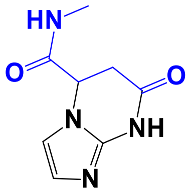

4i

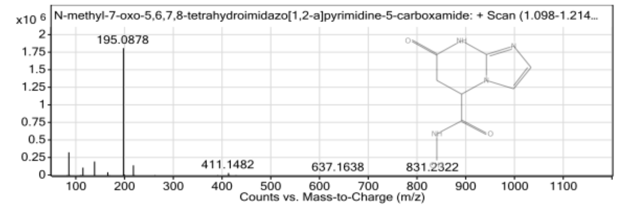

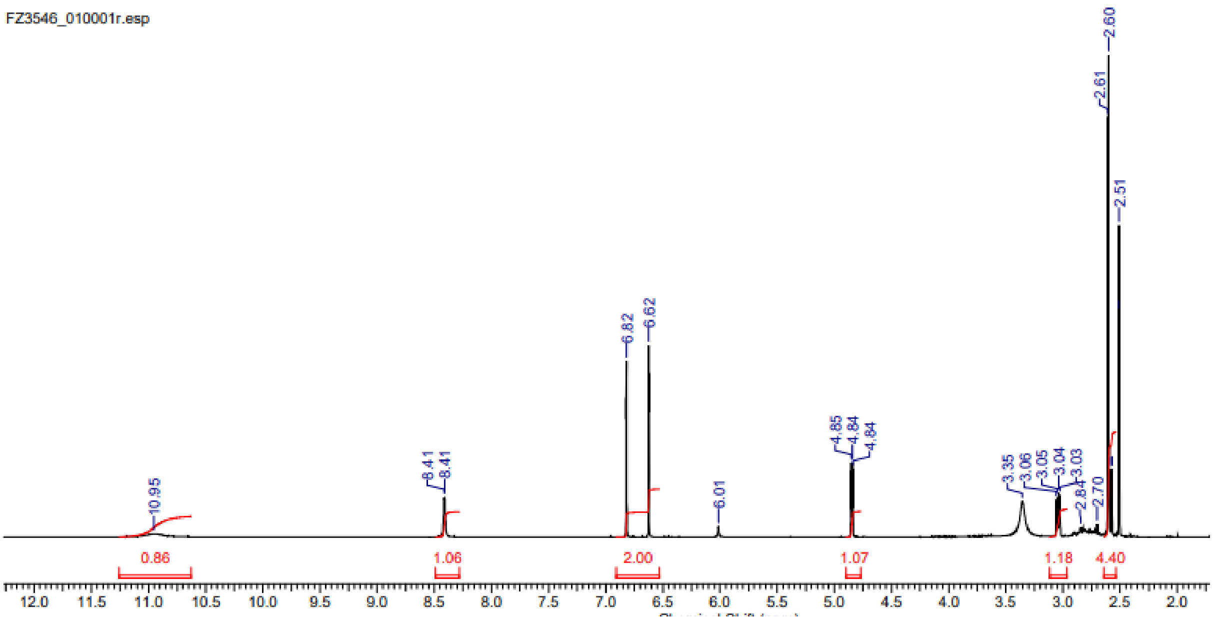

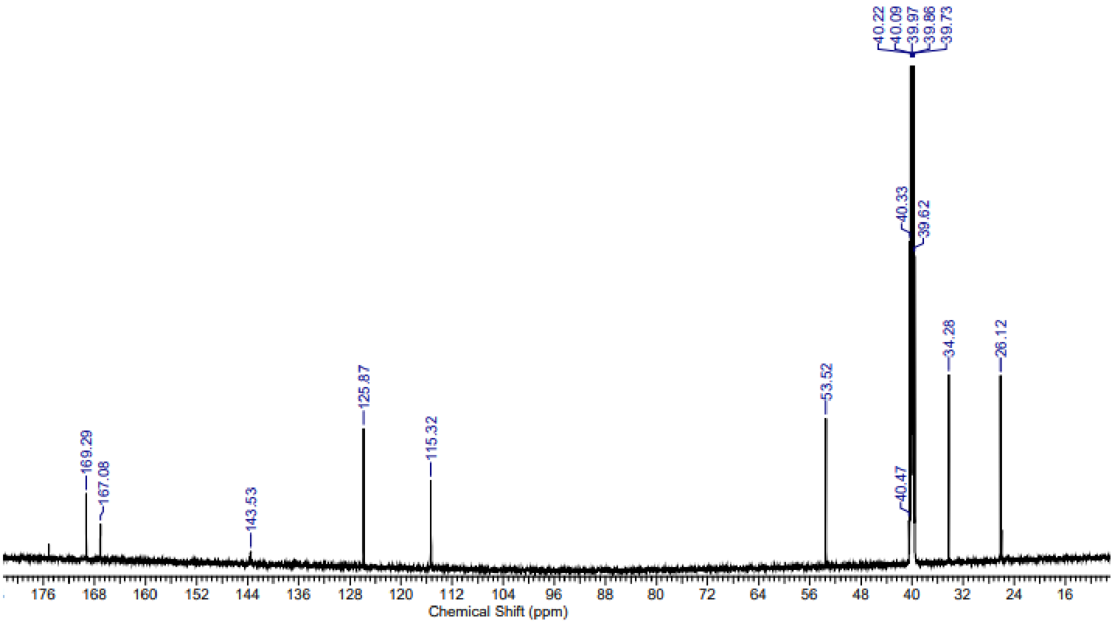

User Chromatograms

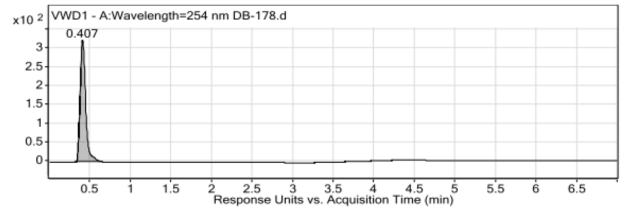

Integration Peak List

Peak	Start	RT	End	Height	Area	Area %
1	0.313	0.407	0.65	323.97	1551.84	100

User Spectra

Fragmentor Voltage	Collision Energy	Ionization Mode
185	0	ESI

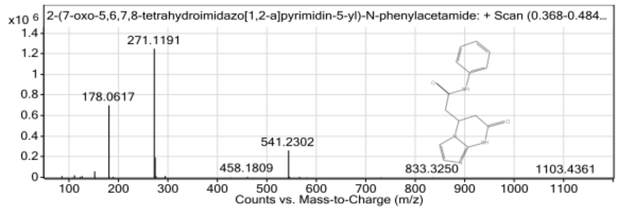

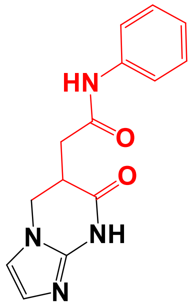

5a

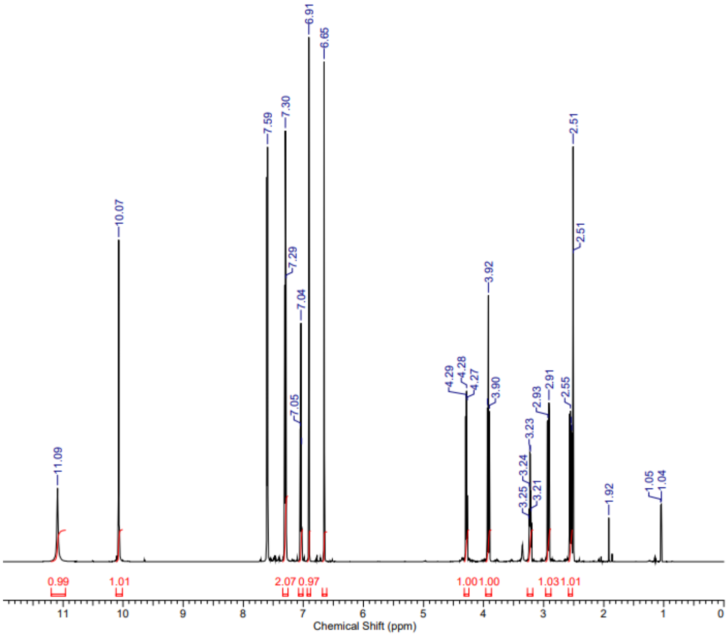

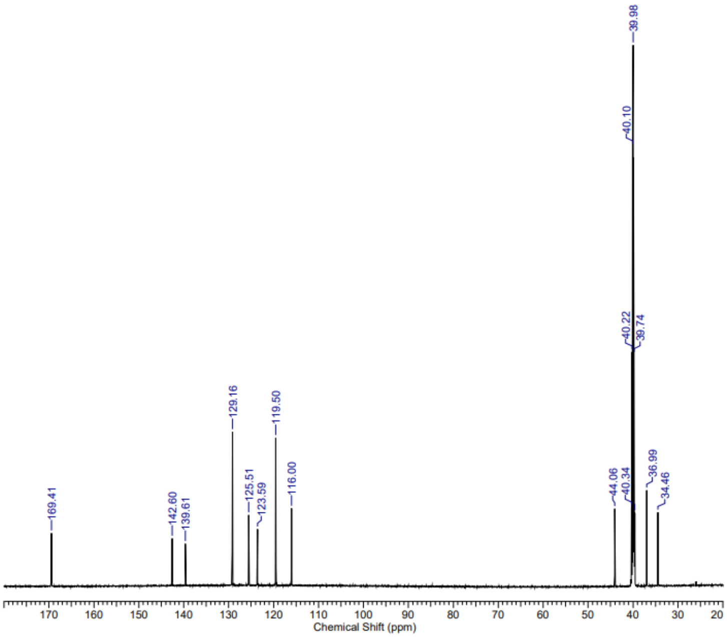

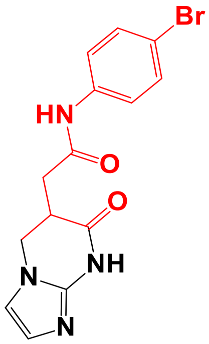

5b

User Chromatograms

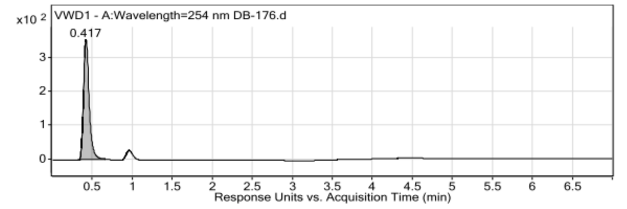

Integration Peak List

Peak	Start	RT	End	Height	Area	Area %
1	0.321	0.417	0.66	356.73	1779.92	100

User Spectra

Fragmentor Voltage	Collision Energy	Ionization Mode
185	0	ESI

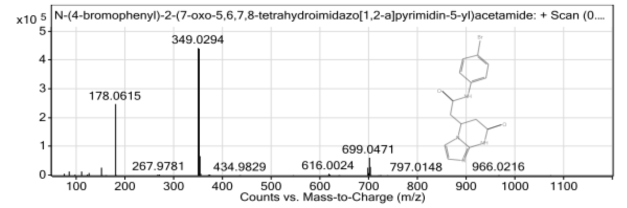

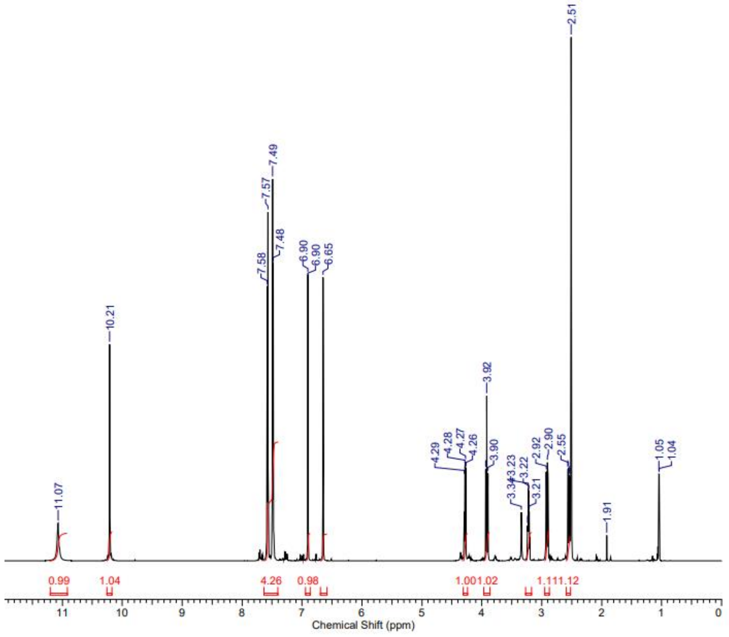

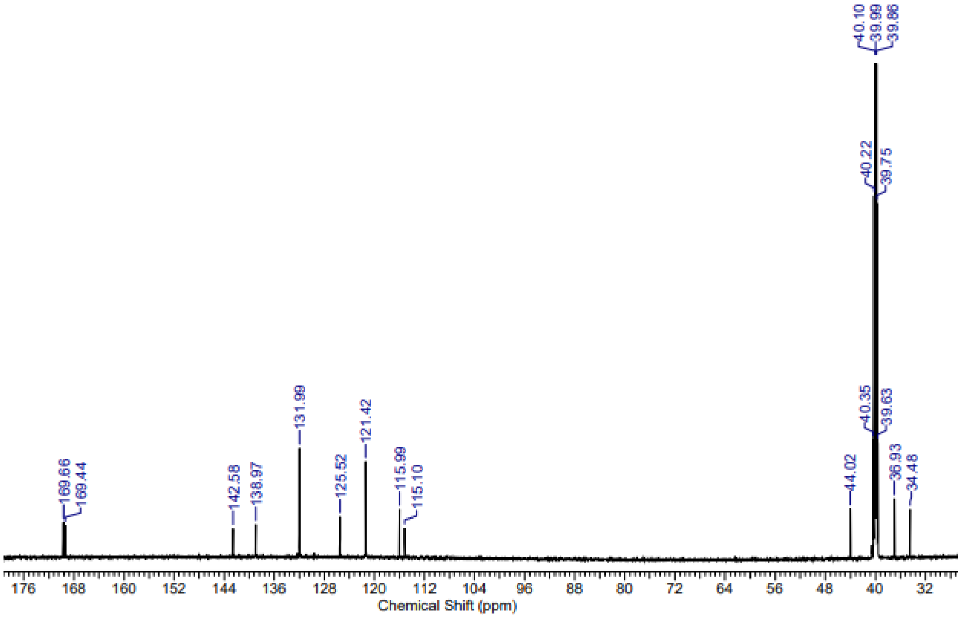

User Chromatograms

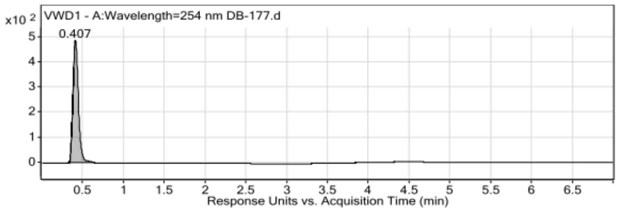

Integration Peak List

Peak	Start	RT	End	Height	Area	Area %
1	0.313	0.407	0.65	488.94	2298.27	100

User Spectra

Fragmentor Voltage	Collision Energy	Ionization Mode
185	0	ESI

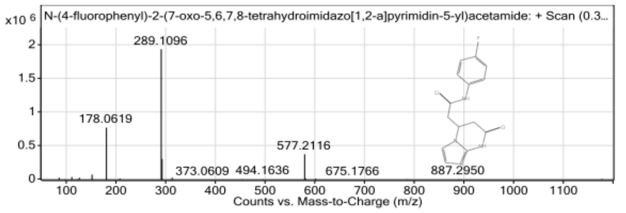

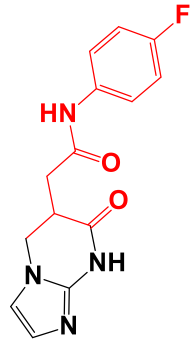

5c

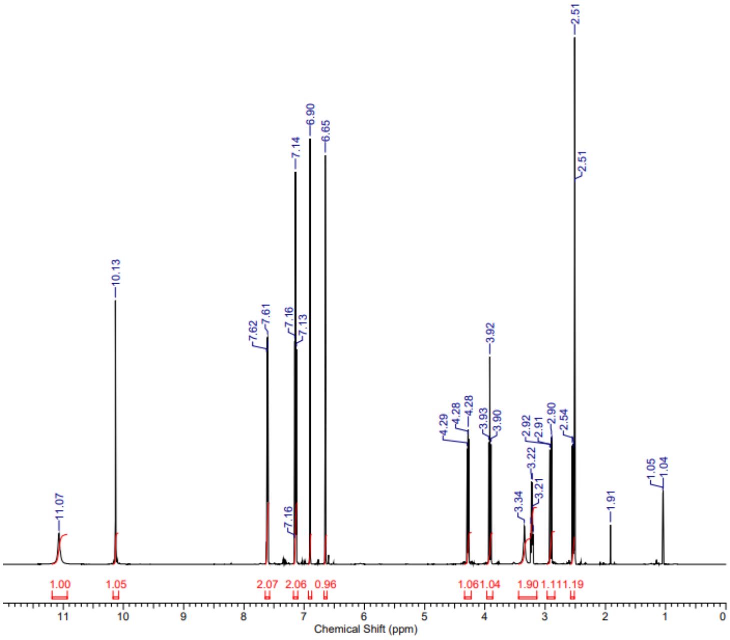

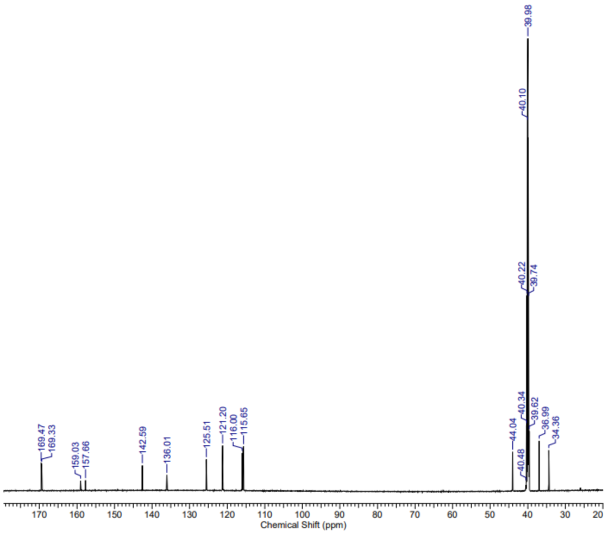
169.47
169.33
159.03
157.66
142.59
136.01
125.51
121.20
116.00
115.65
44.04
40.48
40.34
40.22
40.10
39.98
39.74
39.62
36.99
34.36
170 160 150 140 130 120 110 100 90 80 70 60 50 40 30 20
Chemical Shift (ppm)

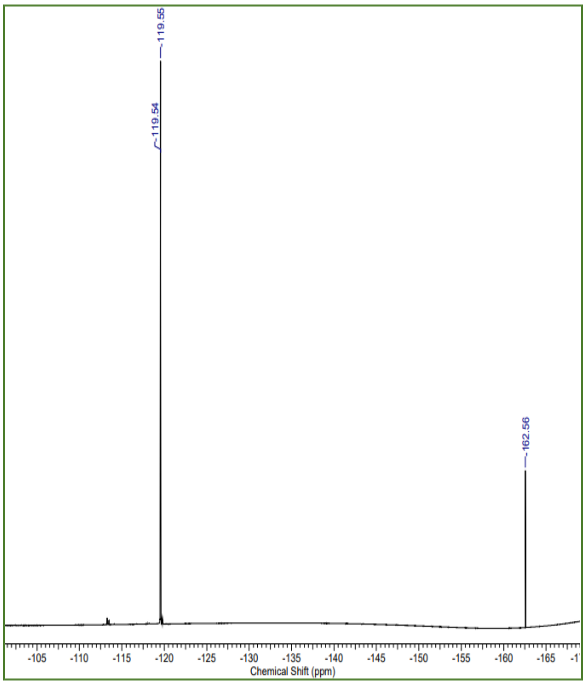
-119.55
-119.54
-162.56
-105 -110 -115 -120 -125 -130 -135 -140 -145 -150 -155 -160 -165
Chemical Shift (ppm)

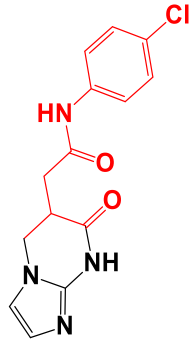

5d

User Chromatograms

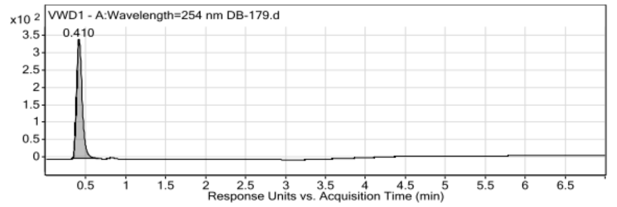

Integration Peak List

Peak	Start	RT	End	Height	Area	Area %
1	0.314	0.41	0.653	343.71	1702.41	100

User Spectra

Fragmentor Voltage	Collision Energy	Ionization Mode
185	0	ESI

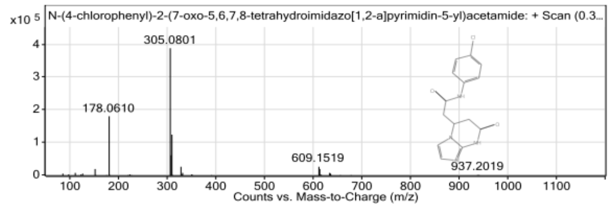

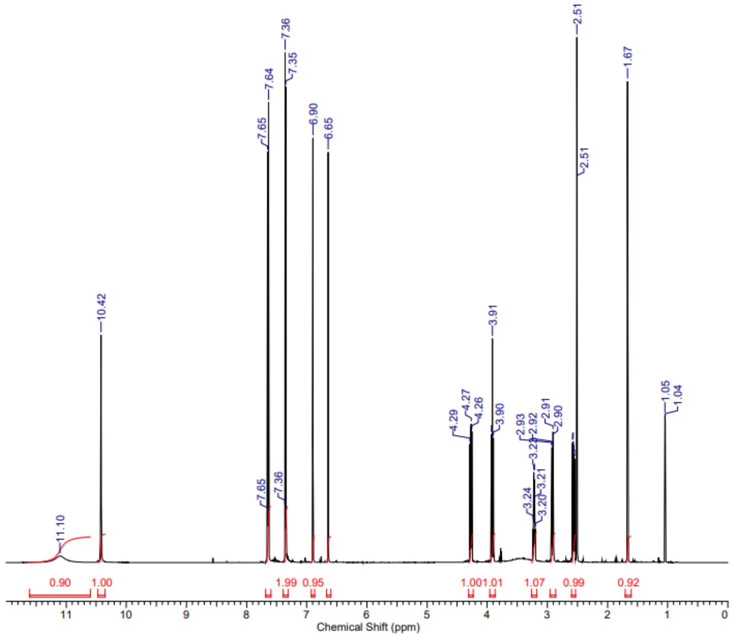

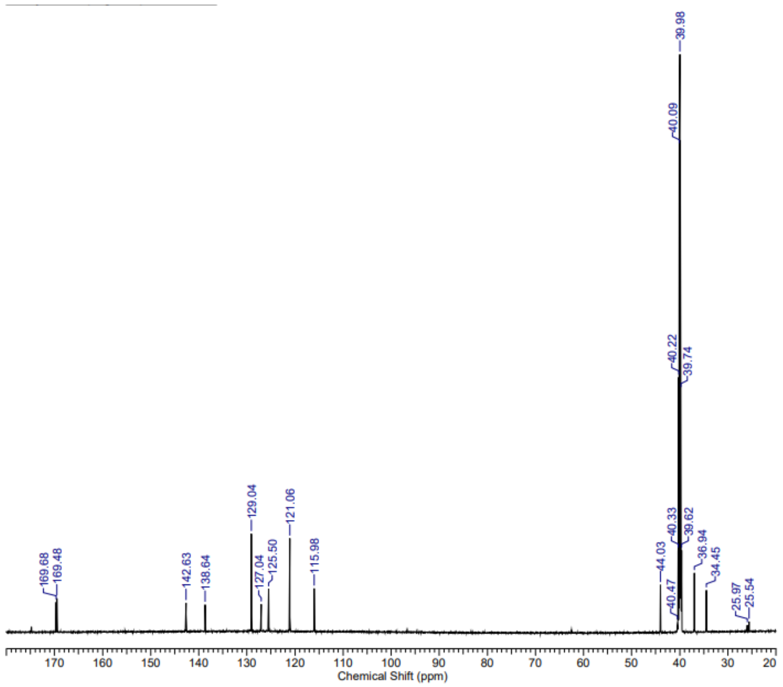

39.98
40.09
40.22
39.74
129.04
121.06
40.33
39.62
36.94
34.45
169.68
169.48
142.63
138.64
127.04
125.50
115.98
44.03
40.47
25.97
25.54
Chemical Shift (ppm)

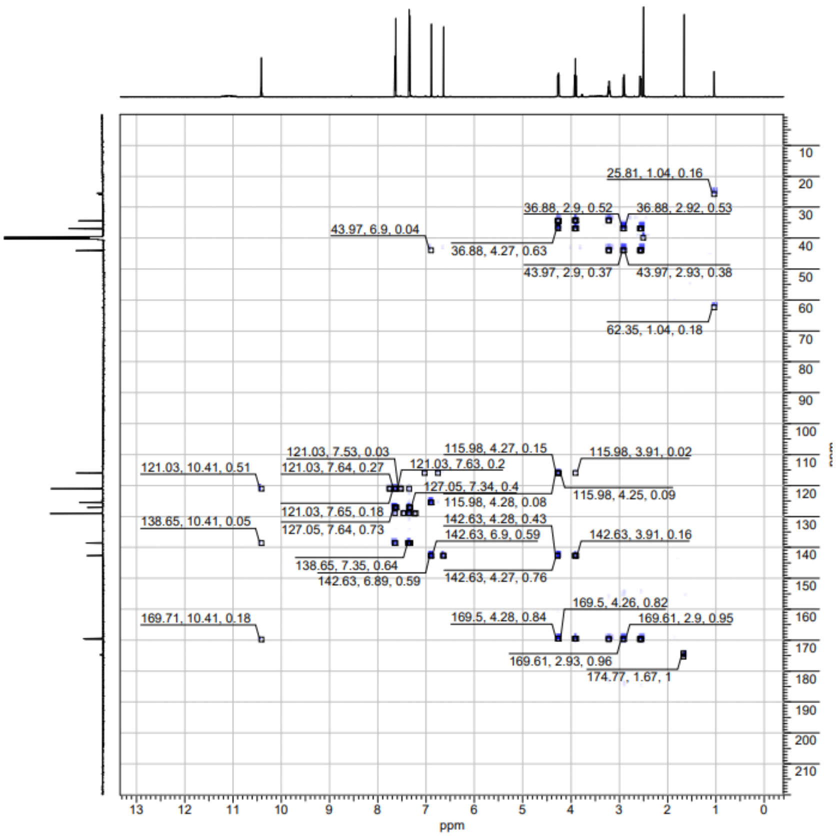

25.81, 1.04, 0.16
36.88, 2.9, 0.52
36.88, 2.92, 0.53
43.97, 6.9, 0.04
36.88, 4.27, 0.63
43.97, 2.9, 0.37
43.97, 2.93, 0.38
62.35, 1.04, 0.18
121.03, 7.53, 0.03
115.98, 4.27, 0.15
115.98, 3.91, 0.02
121.03, 10.41, 0.51
121.03, 7.64, 0.27
121.03, 7.63, 0.2
127.05, 7.34, 0.4
115.98, 4.25, 0.09
115.98, 4.28, 0.08
121.03, 7.65, 0.18
142.63, 4.28, 0.43
138.65, 10.41, 0.05
127.05, 7.64, 0.73
142.63, 6.9, 0.59
142.63, 3.91, 0.16
138.65, 7.35, 0.64
142.63, 6.89, 0.59
142.63, 4.27, 0.76
169.5, 4.26, 0.82
169.71, 10.41, 0.18
169.5, 4.28, 0.84
169.61, 2.9, 0.95
169.61, 2.93, 0.96
174.77, 1.67, 1
ppm

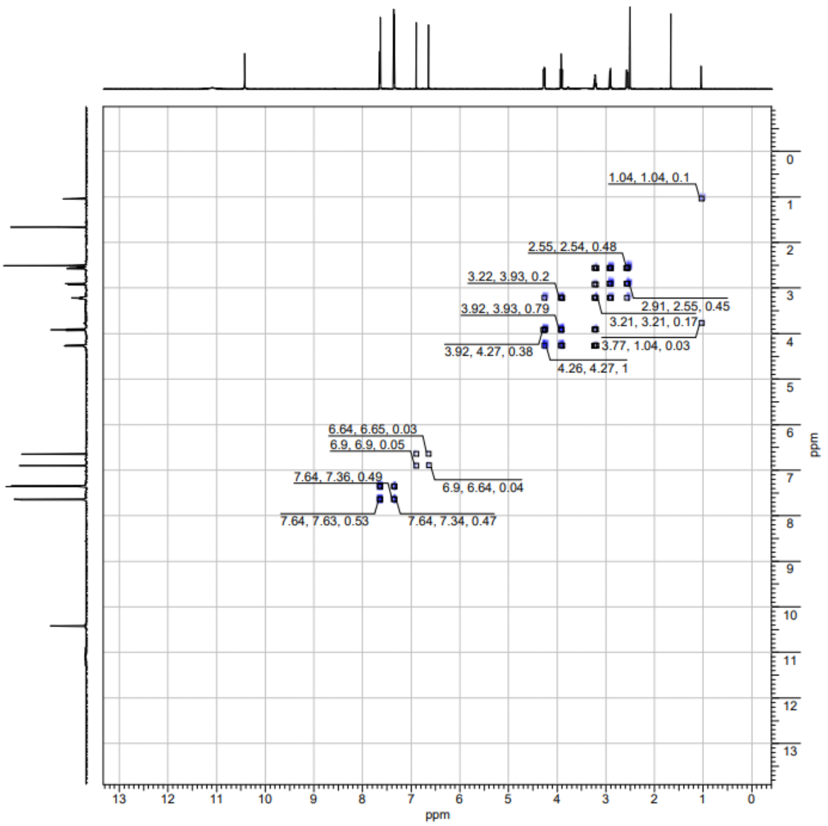

1.04, 1.04, 0.1
2.55, 2.54, 0.48
3.22, 3.93, 0.2
2.91, 2.55, 0.45
3.92, 3.93, 0.79
3.21, 3.21, 0.17
3.92, 4.27, 0.38
3.77, 1.04, 0.03
4.26, 4.27, 1
6.64, 6.65, 0.03
6.9, 6.9, 0.05
7.64, 7.36, 0.49
6.9, 6.64, 0.04
7.64, 7.63, 0.53
7.64, 7.34, 0.47
ppm
ppm

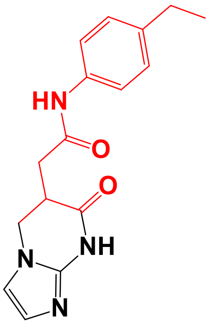

5e

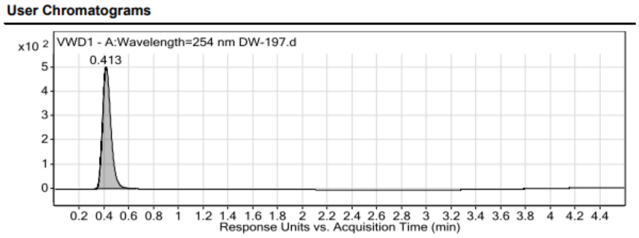

Integration Peak List

Peak	Start	RT	End	Height	Area	Area %
1	0.32	0.413	0.657	506.63	2382.89	100

User Spectra

Fragmentor Voltage	Collision Energy	Ionization Mode
185	0	ESI

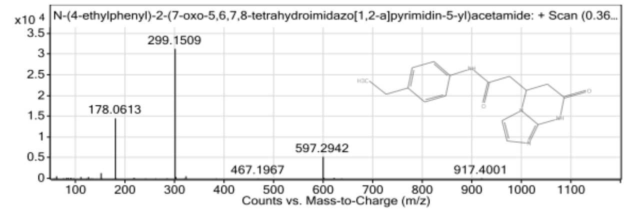

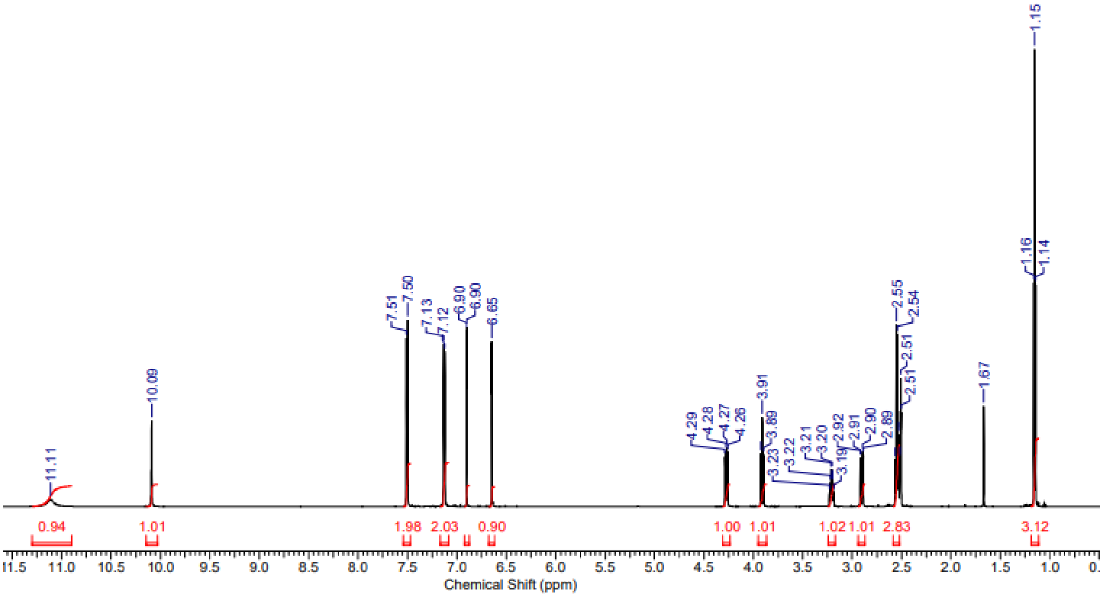

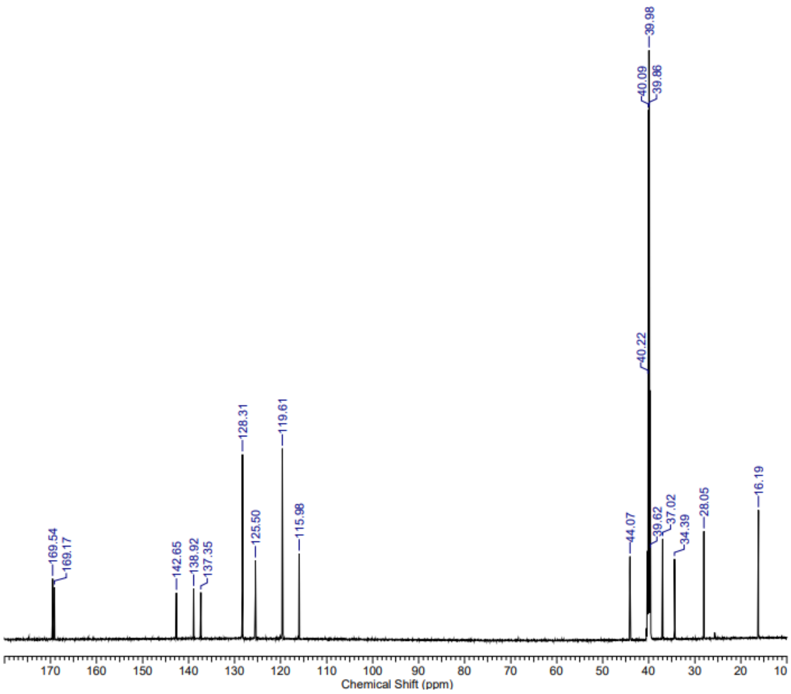

169.54
169.17
142.65
138.92
137.35
128.31
125.50
119.61
115.98
44.07
40.22
40.09
39.98
39.86
39.62
37.02
34.39
28.05
16.19
170 160 150 140 130 120 110 100 90 80 70 60 50 40 30 20 10
Chemical Shift (ppm)
